# A competency addition procedure for constructing minimal, yet maximally informative tests for skill assessment

**DOI:** 10.3758/s13428-026-03121-x

**Published:** 2026-08-03

**Authors:** Pasquale Anselmi, Jürgen Heller, Luca Stefanutti, Egidio Robusto

**Affiliations:** 1 Department of Philosophy, Sociology, Education and Applied Psychology (FISPPA), University of Padova, Via Venezia 14, 35131 Padova, Italy; 2 Department of Psychology, University of Tübingen, Schleichstraße 4, 72076 Tübingen, Germany

**Keywords:** Skill assessment, Test construction, Short tests, Competence structure, Competence model, Problem function

## Abstract

Competence-based test development is a novel method for constructing tests that are as informative as possible about the competence state (the set of skills an individual possesses) underlying item responses. If desired, the tests can also be minimal, meaning that no item can be removed without reducing their informativeness. Consider a set of competencies, each encompassing the skills required to solve a particular item. An individual masters an item if their competence state includes all the skills in the item’s associated competency. A reduct is defined by a competency set that is as informative about individuals’ competence states as a larger set, and from which no competency can be removed without reducing its informativeness. Test development can be based on the reduct, since including only one item for each competency in the reduct yields a test that is as informative as a test that contains additional items and is minimal with respect to this property. This work introduces the competency addition procedure, an iterative method that successively collects competencies until the resulting set forms a reduct. The competencies to be added can be selected either randomly or based on test developer preferences, thereby favoring competencies with more desirable characteristics. The procedure is illustrated in three real-life applications for the assessment of arithmetic skills, involving the construction of a test from scratch, the improvement of an existing test, and the shortening of an existing test.

## Introduction

Competence-based knowledge structure theory (CbKST; Doignon, 1994; Düntsch & Gediga, 1995; Korossy, 1997) allows the identification of the specific attributes (skills, symptoms) possessed by an individual based on their responses to test items. Assessments conducted within this framework span a variety of domains in which items can be meaningfully linked to the attributes required to solve or endorse them. Examples include hard skills (e.g., arithmetic, Anselmi, Stefanutti, de Chiusole, & Robusto, 2017; basic statistics, de Chiusole, Stefanutti, Anselmi, & Robusto, 2018, 2020), soft skills (e.g., collaborative and teamwork skills, Morleo, Anselmi, & Vitanza, 2025; social and emotional skills, Morleo, Vitanza, & Anselmi, 2026), psychological functioning (e.g., depression, Serra, Spoto, Ghisi, & Vidotto, 2017), and neuropsychological functioning (e.g., fluid intelligence, de Chiusole et al., 2026). These assessments may also differ substantially in the number of attributes and items involved. For instance, CoTeSt (Collaborative and Teamwork Skill test) is a 20-item test aimed at assessing which of six collaborative and teamwork skills teachers possess (Morleo et al., 2025), whereas MatriKS 4–11 is an adaptive test consisting of 38 Raven-like matrices and aimed at assessing which of the nine skills required to solve them children possess (de Chiusole et al., 2026).

The present work focuses on the educational assessment context. Accordingly, the attributes under consideration are referred to as “skills”, and the items are described as solved or not because responses are typically interpreted as correct or incorrect. In the clinical assessment context, the attributes may instead be referred to as “symptoms” or “diagnostic criteria”, and the items may be described as endorsed or not because responses are typically not interpreted in terms of correctness. The present work further considers conjunctive tests, in which an item is expected to be solved or endorsed if all the attributes associated with it are possessed. This type of test is especially appropriate in educational settings, where solving an item usually requires a series of steps, all of which must be carried out correctly. However, the proposed methodology also applies to the development of disjunctive tests, in which an item is expected to be solved or endorsed if at least one of the attributes associated with it is possessed. Disjunctive tests may be more suitable in some clinical contexts, where a disorder may be diagnosed based on the presence of only one of its symptoms (Anselmi, Heller, Stefanutti, & Robusto, 2025a; Roussos, Templin, & Henson, 2007; Templin & Henson, 2006).

Competence-based test development (CbTD; Anselmi, Heller, Stefanutti, & Robusto, 2022, 2024b) is a recent and innovative method for developing tests that are as informative as possible about the specific skills that an individual possesses. If desired, the tests can also be minimal, meaning that the exclusion of any item would reduce their informativeness. CbTD can be used in various test development contexts, including constructing tests from scratch, improving existing tests, and shortening them. For example, Anselmi, Heller, Stefanutti, Robusto, and Barillari (2025b) constructed a test for the assessment of students’ mathematical formulation, improved a booklet from the TIMSS (Trends in International Mathematics and Science Study; https://timss.bc.edu/) 2003 eighth-grade mathematics test, and shortened Tatsuoka ’s (1990) fraction subtraction test. Dörrbecker and Heller (2025) shortened the subtraction subtest of the Heidelberger Rechentest 1–4 (Haffner, Baro, Parzer, & Resch, 2005), which is one of the most commonly used German elementary school speed numeracy tests.

CbTD develops from CbKST, inheriting mathematical notation, basic concepts, and results on test properties that allow for uniquely uncovering the set of skills an individual possesses (referred to as the *competence state*) from the set of items the individual masters (referred to as the *knowledge state*; Heller, Anselmi, Stefanutti, & Robusto, 2017; Heller, Stefanutti, Anselmi, & Robusto, 2015, 2016). Other concepts have been inspired by rough set theory (RST; Pawlak, 1982, 1991), particularly by the work of Yao and Zhao (2009), and Yao, Zhao, Wang, and Han (2006, 2008). Section “Background” describes CbTD and some of its concepts. There are strong links between CbKST and cognitive diagnostic models (CDMs; DiBello, Roussos, & Stout, 2007; Rupp, Templin, & Henson, 2010; Tatsuoka, 1990), which provide another framework for identifying skills from item responses. These links were first established by Heller et al. (2015, 2016) and further elaborated by Anselmi, Noventa, and Heller (2024a), Heller (2022), Noventa, Heller, and Kelava (2024), and Stefanutti, Anselmi, de Chiusole, Spoto, and Heller (2025). Thus, CbTD can be utilized for test development within both CbKST and CDMs.

Consider a *competency set*, that is, a nonempty family of nonempty subsets of skills. Each subset is called a *competency* and identifies the skills required to solve an item that exists or can be constructed. An individual masters the item if they possess all the skills included in the item’s associated competency. A fundamental concept in CbTD is that of a *reduct*, which is defined by a competency set that is as informative about individuals’ competence states as a larger competency set, and from which no competency can be removed without reducing its informativeness. Test development may be based on a reduct. If for each competency in the reduct there is an item that requires all the skills in that competency for its solution, a conjunctive test is obtained that is as informative as a test that contains additional items. If the test contains only one item for each competency in the reduct, the test is also minimal. Constructing a reduct is a crucial step in test development within the proposed approach. Specifically, shortening an existing test involves selecting one available item for each competency in the reduct, whereas constructing a test from scratch or improving an existing test involves creating one item for each competency in the reduct.

Anselmi et al. (2022) proposed the *competency deletion procedure* (CDP), an iterative method that successively deletes one competency at a time from the full competency set until the remaining set forms a reduct. Anselmi et al. (2024b) described a variant of the procedure that allows the removal of multiple competencies at once, thereby reducing the number of iterations needed to obtain the reduct. The authors also illustrated a simple way to incorporate test developer preferences (e.g., preferring items that require fewer skills over those that require more) into the CDP. However, both versions of the CDP will not be very efficient when the target reduct has small cardinality and many competencies must be eliminated from the full competency set to obtain the reduct.

This work proposes the *competency addition procedure* (CAP), an iterative method that successively collects competencies until the resulting set forms a reduct. The CAP is expected to be more efficient than the CDP when the target reduct has a small cardinality. Section “Partial reduct and core” introduces some new concepts that form the basis of the CAP as outlined in Section “Competency addition procedure”. Section “Incorporating preferences into the competency addition procedure” illustrates how test developer preferences can be incorporated into the CAP. Section “Real-life applications” presents applications of the CAP to the construction, improvement, and shortening of tests for the assessment of arithmetic skills. Section “Final remarks” elaborates on extensions of the presented approach and outlines directions for future research. The appendix provides a simplified overview of basic concepts related to CbTD.

## Background

The concepts described in this section can be found in the literature on CbTD (Anselmi et al., 2022, 2025b, 2024b), CbKST (e.g., Doignon, 1994; Düntsch & Gediga, 1995; Gediga, & Düntsch, 2002; Heller, Ünlü, & Albert, 2013; Heller et al., 2015, 2016, 2017; Heller, & Stefanutti, 2024; Korossy, 1997, 1999), and RST (e.g., Pawlak, 1982, 1991; Yao et al., 2006; Yao and Zhao, 2008), to which the interested reader is referred for a more detailed treatment.

### Competence models

Let *S* be a nonempty finite collection of skills to be assessed. A *competence structure* defined on *S* is a family $\mathcal {C}$ of subsets of *S* containing at least ∅ and *S* (Korossy, 1997, 1999). Each subset $C\in \mathcal {C}$ is called a *competence state* and identifies all the skills that an individual possesses. Consider a nonempty finite collection *Q* of dichotomous items, each of which can be solved using some of the skills in *S*. In what follows, *Q* is sometimes called a *test*. In the tests considered in this work, each item is associated with a specific subset of skills relevant to solving it. The link between the items in *Q* and the skills in *S* that are relevant to their solution is formally established through a *competence model*, which is a quadruple $(Q,S,\mathcal {C},\mu )$, where $\mu :Q \rightarrow 2^{\left( 2^S\right) }$ ($2^X$ denotes the set of all subsets of *X*) is a mapping associating a nonempty collection of nonempty, pairwise incomparable (with respect to set inclusion) subsets of *S* to each item q∈Q (Heller et al., 2017). Each subset $T \in \mu (q)$ is called a *competency for q* and represents a strategy for solving *q*. A competence model $(Q,S,\mathcal {C},\mu )$ is said to be *conjunctive* when every item in *Q* has a single solution strategy consisting in the application of one or more skills and it is said to be *disjunctive* when every item in *Q* has one or more solution strategies, each consisting in the application of a single skill. In what follows, a test *Q* is called “conjunctive” or “disjunctive” when one of these properties holds for the competence model that includes it. This work focuses on conjunctive tests. However, as illustrated in Section “Final remarks”, the presented methodology also applies to disjunctive tests.

It is reasonable to assume that an individual masters a certain item if they possess all the skills that constitute at least one strategy for solving it. That is, if *C* is the competence state of the individual, then item q∈Q is mastered by the individual if there is a competency $T \in \mu (q)$ such that T⊆C. The connection between possessed skills and mastered items is formally established through a *problem function*, which is a mapping $p:\mathcal {C} \rightarrow 2^{Q}$ induced by a competence model $(Q, S, \mathcal {C}, \mu )$ and defined by

$$\begin{aligned} p (C) = \{q \in Q : \text{ there } \text{ is } \text{ a } T \in \mu (q) \text{ such } \text{ that } T \subseteq C\} \end{aligned}$$

for all $C \in \mathcal {C}$ (Heller et al., 2013). Specifically, the problem function associates each competence state $C \in \mathcal {C}$ with the collection *p*(*C*) of all items in *Q* that are mastered by an individual possessing the skills in *C*; this collection is the *knowledge state* delineated by the competence state *C*. The family $p(\mathcal {C}) = \{p(C):C \in \mathcal {C}\}$ of all knowledge states delineated by all competence states in $\mathcal {C}$ is the *knowledge structure* delineated by the competence structure $\mathcal {C}$.

The following example illustrates the concepts introduced so far. For competence states, competencies, and knowledge states, *xy* is used as a shorthand notation for the set $\{x,y\}$.

#### Example 1

Consider the two conjunctive competence models $M_1 = (Q,S,\mathcal {C},\mu _1)$ and $M_2 = (Q,S,\mathcal {C},\mu _2)$, with $Q = \{1,2,3,4\}$, $S = \{a,b,c,d\}$, $\mathcal {C} = \{\emptyset ,b,ab,ac,abc,bcd,abcd\}$, and the mappings $\mu _1$ and $\mu _2$ defined by

$$ \mu _1 (1) = \{b\},~ \mu _1 (2) = \{ab\},~ \mu _1 (3) = \{ac\},~ \mu _1 (4) = \{cd\}, $$

and

$$ \mu _2 (1) = \{a\},~ \mu _2 (2) = \{b\},~ \mu _2 (3) = \{ad\},~ \mu _2 (4) = \{bcd\}. $$

According to $M_1$, for instance, item 1 requires skill *b* to be solved, and item 2 requires both skills *a* and *b*. The knowledge states delineated by the competence states in $\mathcal {C}$ via $M_1$ are

$$\begin{aligned}&p_1 (\emptyset ) = \emptyset ,~ p_1 (b) = 1,~ p_1 (ab) = 12,~ p_1 (ac) = 3,\\&\qquad ~ p_1 (abc) = 123,~ p_1 (bcd) = 14,~ p_1 (abcd) = 1234. \end{aligned}$$

For instance, competence state *bcd* delineates knowledge state 14 because it contains all the skills required by items 1 and 4 but lacks skill *a*, which is required by items 2 and 3. Thus, an individual possessing skills *b*, *c*, and *d* masters items 1 and 4. The delineated knowledge structure is $p_1(\mathcal {C}) = \{\emptyset ,1,3,12,14,123,1234\}$. The knowledge states delineated by the competence states in $\mathcal {C}$ via $M_2$ are

$$\begin{aligned}&p_2 (\emptyset ) = \emptyset ,~ p_2 (b) = 2,~ p_2 (ab) = 12,~ p_2 (ac) = 1,\\&\qquad ~ p_2 (abc) = 12,~ p_2 (bcd) = 24,~ p_2 (abcd) = 1234, \end{aligned}$$

and the delineated knowledge structure is $p_2(\mathcal {C}) = \{\emptyset ,1,2,12,24,1234\}$.

We now describe competence models and properties that are particularly relevant to the test-development approach outlined in this work. The notions of *domain restriction* and *domain extension* of a competence model $(Q,S,\mathcal {C},\mu )$ refer to the removal of items from and the addition of items to the test *Q*, respectively (Anselmi et al., 2022; Heller et al., 2017). Specifically, a domain restriction of $(Q,S,\mathcal {C},\mu )$ is a competence model $(Q^-,S,\mathcal {C},\mu ^-)$ that keeps only some of the items from *Q* ($\emptyset \subset Q^- \subset Q$), whereas a domain extension of $(Q,S,\mathcal {C},\mu )$ is a competence model $(Q^+,S,\mathcal {C},\mu ^+)$ that contains all items in *Q* plus additional ones ($Q \subset Q^+$). Domain restrictions and domain extensions are relevant to shortening (Section “Shortening an existing test”) and improving (Section “Improving an existing test”) existing tests.

We now introduce the concept of test informativeness, which is the extent to which competence states can be uncovered from knowledge states. In general, the fewer distinct competence states in $\mathcal {C}$ that delineate the same knowledge state, the lower the uncertainty about an individual’s competence state and, consequently, the higher the test informativeness. A competence model $(Q,S,\mathcal {C},\mu )$ is said to be *fully informative* if the problem function *p* induced by $(Q,S,\mathcal {C},\mu )$ is injective, that is, if there are no two distinct competence states in $\mathcal {C}$ that delineate the same knowledge state. In a fully informative competence model, any competence state can be uniquely uncovered from the knowledge state. A competence model $M_1 = (Q_1,S,\mathcal {C},\mu _1)$ is said to be *more informative* than a competence model $M_2 = (Q_2,S,\mathcal {C},\mu _2)$ if there are distinct competence states in $\mathcal {C}$ that delineate the same knowledge state via $M_2$ and not via $M_1$, but not vice versa. If a competence model is more informative than another, then it allows for distinguishing more competence states from the knowledge states. Competence models $M_1$ and $M_2$ are said to be *equally informative* if, whenever any two distinct competence states in $\mathcal {C}$ delineate the same knowledge state via one of the two competence models, this also holds for the other competence model. Equally informative competence models allow for distinguishing the same competence states from the knowledge states. In some cases, one may prefer shorter tests among those which are equally informative. A competence model $(Q,S,\mathcal {C},\mu )$ is said to be *minimal* (*irreducible* in Anselmi et al., 2022) if it is more informative than any domain restriction of it. In a minimal competence model, all items in *Q* are necessary to distinguish the pairs of competence states that are originally distinguished. In the following, the tests are referred to as “fully informative”, “more informative”, “equally informative”, and “minimal” whenever these properties hold for the competence models including them.

#### Example 2

Consider the competence models $$M_1$$ and $$M_2$$ and their corresponding problem functions $$p_1$$ and $$p_2$$ defined in Example 1. $$M_1$$ is fully informative because $$p_1$$ is injective: no two distinct competence states in $$\mathcal {C}$$ delineate the same knowledge state ($$p_1(C) \ne p_1(C')$$ for all $$C,C' \in \mathcal {C}$$ with $C\neq C^{\prime }$). $$M_2$$ is not fully informative because $$p_2$$ is not injective: competence states *ab* and *abc* in $$\mathcal {C}$$ delineate the same knowledge state, namely 12 ($$p_2(ab) = p_2(abc)=12$$). Thus, $$M_1$$ allows for uniquely uncovering the competence state of any individual from a given knowledge state, whereas $$M_2$$ does not. Specifically, in $$M_2$$, if the knowledge state of an individual is 12, then their competence state can be either *ab* or *abc*, whereas this ambiguity does not arise in $$M_1$$. This in turn implies that $$M_1$$ is more informative than $$M_2$$. $$M_1$$ is minimal. Competence states ∅ and *b* delineate distinct knowledge states in $$M_1$$ ($$p_1(\emptyset ) = \emptyset \text {;} p_1(b) = 1$$), but the same knowledge state in the domain restriction $$M_1^- = (Q^-,S,\mathcal {C},\mu _1^-)$$ of $$M_1$$, with $$Q^- = \{2,3,4\}$$ ($$p^-_1(\emptyset ) = p^-_1(b) = \emptyset $$). Analogous cases are observed in all other possible domain restrictions of $$M_1$$, meaning that $$M_1$$ is more informative than any of its domain restrictions. $$M_2$$ is not minimal. Consider the domain restriction $$M_2^- = (Q^-,S,\mathcal {C},\mu _2^-)$$ of $$M_2$$, with $$Q^- = \{1,2,4\}$$. The knowledge states delineated by the competence structure $$\mathcal {C}$$ via $$M_2^-$$ are

$$\begin{aligned}&p^-_2 (\emptyset ) = \emptyset ,~ p^-_2 (b) = 2,~ p^-_2 (ab) = 12,~ p^-_2 (ac) = 1,\\&\qquad ~ p^-_2 (abc) = 12,~ p^-_2 (bcd) = 24,~ p^-_2 (abcd) = 124. \end{aligned}$$

Apart from *ab* and *abc*, which delineate the same knowledge state via $M_2$, there are no additional distinct competence states in $\mathcal {C}$ that delineate the same knowledge state via $M_2^-$. Thus, $M_2$ and $M^-_2$ are equally informative. Item 3 can be removed from the test corresponding to $M_2$ without reducing its informativeness, whereas removing any item from the test corresponding to $M_1$ would reduce that test’s informativeness.

The competence model $(Q,S,\mathcal {C},\mu )$ is a theoretical model of the association between items and skills, as expressed by the mapping μ. Various methods can be used to determine whether the items actually require the assumed skills (Ketabi, Alavi, & Ravand, 2021, Ravand, & Baghaei, 2020). Experts can be presented with the test and a list of skills and asked to match each item to the skills required to solve it. Test-takers can be instructed to think aloud while responding to the items or to explain their thinking afterward. Finally, the competence model can be validated by fitting a probabilistic model, such as the basic local independence model (BLIM; Falmagne & Doignon, [Bibr CR15][Bibr CR16]) or the competence-based local independence model (CBLIM; Heller et al., 2015, 2016), to empirical data collected from test-takers.

The problem function *p* induced by $(Q,S,\mathcal {C},\mu )$ deterministically specifies which items are mastered by an individual who possesses the skills in *C*. In practical applications, however, there need not be a perfect correspondence between the items an individual masters (i.e., the knowledge state delineated by the individual’s competence state) and the items that individual actually solves (i.e., the individual’s response pattern). For example, an individual may fail to solve an item they master due to inattention, or may solve an item they do not master by guessing the correct answer (e.g., when responding to a multiple-choice item). In the educational assessment context, where responses are typically interpreted as correct or incorrect, these two conditions are referred to as a careless error and a lucky guess, respectively. In the clinical assessment context, where responses are typically interpreted in terms of endorsement, a negative response (e.g., no, false) from a person who should have answered positively (e.g., yes, true) is termed a false negative, whereas a positive response from a person who should have answered negatively is termed a false positive. By fitting the BLIM or the CBLIM to empirical data, careless error and lucky guess probabilities are obtained for each item and, based on these probabilities, an individual’s knowledge state is inferred from their response pattern. The individual’s competence state is then deterministically derived from the knowledge state. If the competence model $(Q,S,\mathcal {C},\mu )$ is fully informative, then for any inferred knowledge state, there is a unique competence state delineating it.

### Discernibility systems, simplifications, and reducts

Consider a set *S* of skills and a competence structure $$\mathcal {C}$$ on *S*. This section explores the possibility of distinguishing pairs of competence states in $$\mathcal {C}$$ by subsets of *S*, which is necessary for uniquely identifying an individual’s competence state from their knowledge state. Here, distinguishing the pair $$\{C,C'\}$$ means distinguishing between the competence states *C* and $C^{\prime }$. The notion of a discernibility system serves this purpose. Formally, a *discernibility system* is a quadruple $$(S,\mathcal {D},\mathcal {T},\delta )$$, where *S* is a nonempty finite collection of skills,  is a nonempty collection of unordered pairs $$\{C,C'\}$$ of competence states in $$\mathcal {C}$$ ( denotes the forming of unordered pairs), $$\mathcal {T}\subseteq 2^S\setminus \{\emptyset \}$$ is a nonempty collection of nonempty subsets *T* of *S* (\ denotes set difference), each of which is interpreted as a competency, and $$\delta :\mathcal {D} \rightarrow 2^{\mathcal {T}}$$ is a mapping defined by

1 $$\begin{aligned} \delta (C,C') = \{T \in \mathcal {T}: (T\subseteq C \wedge T\not \subseteq C') \vee (T\subseteq C' \wedge T\not \subseteq C)\} \end{aligned}$$

for all $$\{C,C'\} \in \mathcal {D}$$ ($\delta (C,C^{\prime })$ is a short form for $$\delta (\{C,C'\})$$). The mapping δ is called the *discernibility function*. The collection $\delta (C,C^{\prime })$ is the *(competency) bag* of the pair $$\{C,C'\}$$ and contains all competencies that are included in exactly one of the two competence states. In other words, a competency $$T \in \mathcal {T}$$ belongs to $\delta (C,C^{\prime })$ if it is a subset of *C* and not of $C^{\prime }$ $$(T\subseteq C \wedge T\not \subseteq C')$$, or a subset of $C^{\prime }$ and not of *C* $$(T\subseteq C' \wedge T\not \subseteq C)$$. Every competency $$T \in \delta (C,C')$$ is sufficient for distinguishing the pair $$\{C,C'\}$$, and the latter can be distinguished if and only if its bag $\delta (C,C^{\prime })$ is nonempty (i.e., $$\delta (C,C') \ne \emptyset $$).

**Table 1:** Discernibility functions $\delta , \delta _1, \delta _2$, and $\delta _3$ for the four discernibility systems in Example 3

$$\{C,C'\} \in \mathcal {D}$$	$\delta (C,C^{\prime })$	$$\delta _1(C,C')$$	$$\delta _2(C,C')$$	$$\delta _3(C,C')$$
$\{\emptyset ,b\}$	∅	∅	∅	∅
$\{ab,ad\}$	$\{ab\}$	$\{ab\}$	$\{ab\}$	$\{ab\}$
$\{ac,bd\}$	$\{a,c,bd\}$	$\{a\}$	$\{c\}$	$\{bd\}$
$\{bd,bcd\}$	$\{c,cd,bcd\}$	$\{cd\}$	$\{c\}$	∅
$\{abc,acd\}$	$\{ab,cd,acd\}$	$\{ab,cd\}$	$\{ab,acd\}$	$\{ab,acd\}$

In this work, particular interest is given to subsets of $$\mathcal {T}$$ that distinguish the same pairs of competence states in $$\mathcal {D}$$ as $$\mathcal {T}$$. The following notions are relevant in this respect. A *simplification* of the discernibility system $$(S,\mathcal {D},\mathcal {T},\delta )$$ is a discernibility system $$(S,\mathcal {D},\mathcal {T}^\diamond ,\delta ^\diamond )$$ such that $$\mathcal {T}^\diamond \subseteq \mathcal {T}$$ and, for each pair $$\{C,C'\}\in \mathcal {D}$$, $$\delta ^\diamond (C,C') = \delta (C,C') \cap \mathcal {T}^\diamond $$, with $$\delta ^\diamond (C,C')$$ being nonempty whenever $\delta (C,C^{\prime })$ is nonempty. A *reduct* of the discernibility system $$(S,\mathcal {D},\mathcal {T},\delta )$$ is a simplification $$(S,\mathcal {D},\mathcal {T}^\diamond ,\delta ^\diamond )$$ of $$(S,\mathcal {D},\mathcal {T},\delta )$$ such that, for each $$T \in \mathcal {T}^\diamond $$, there is some pair $$\{C,C'\} \in \mathcal {D}$$ satisfying $$\delta ^\diamond (C, C') = \{T\}$$.^1^ This condition means that competency *T* is necessary and sufficient to distinguish *C* and $C^{\prime }$. In a simplification $$(S,\mathcal {D},\mathcal {T}^\diamond ,\delta ^\diamond )$$ of $$(S,\mathcal {D},\mathcal {T},\delta )$$, $$\mathcal {T}^\diamond $$ is a subset of $$\mathcal {T}$$ that allows for distinguishing exactly the same pairs of competence states as $$\mathcal {T}$$. In a reduct $$(S,\mathcal {D},\mathcal {T}^\diamond ,\delta ^\diamond )$$ of $$(S,\mathcal {D},\mathcal {T},\delta )$$, $$\mathcal {T}^\diamond $$ is a minimal (with respect to set inclusion) subset of $$\mathcal {T}$$ that allows for distinguishing exactly the same pairs of competence states as $$\mathcal {T}$$ (i.e., none of the competencies in $$\mathcal {T}^\diamond $$ can be removed without emptying some bag). Thus, the competencies in a simplification are sufficient to distinguish the same pairs of competence states as the original discernibility system, and each competency in a reduct is also necessary.

Given *S* and $\mathcal {D}$, δ is uniquely determined by $\mathcal {T}$. Thus, the discernibility system $(S,\mathcal {D},\mathcal {T},\delta )$ is fully determined by its competency set $\mathcal {T}$. Accordingly, when *S* and $\mathcal {D}$ are clear from the context, we may simply refer to $(S,\mathcal {D},\mathcal {T},\delta )$ as the discernibility system with competency set $\mathcal {T}$. Additionally, we may say that $(S,\mathcal {D},\mathcal {T},\delta )$ contains certain competencies to mean that $\mathcal {T}$ contains those competencies.

#### Example 3

Consider the discernibility system $(S,\mathcal {D},\mathcal {T},\delta )$, where $S = \{a,b,c,d\}$, $\mathcal {D} = \{\{\emptyset ,b\},\{ab,ad\},\{ac,bd\},\{bd,bcd\},\{abc,acd\}\}$, $\mathcal {T}= \{a,c,ab,bd,cd,acd,bcd\}$, and where the discernibility function δ is defined as in Table 1. Let us consider, for instance, pair $\{abc,acd\}$ in $\mathcal {D}$. Competency *ab* in $\mathcal {T}$ is a subset of *abc* but not of *acd*, competencies *cd* and *acd* are subsets of *acd* but not of *abc*, competencies *a* and *c* are subsets of both *abc* and *acd*, and competencies *bd* and *bcd* are subsets of neither *abc* nor *acd*. Thus, according to Eq. 1, the bag of pair $\{abc,acd\}$ is $\delta (abc,acd) = \{ab,cd,acd\}$, and any of the three competencies in it is sufficient for distinguishing $\{abc,acd\}$. For instance, an item that requires both skills *a* and *b* to be solved is sufficient for determining whether an individual possesses skills *a*, *b*, and *c* or skills *a*, *c*, and *d*. The bag of pair $\{\emptyset ,b\}$ is empty ($\delta (\emptyset ,b)=\emptyset$), meaning that no competency in $\mathcal {T}$ distinguishes $\{\emptyset ,b\}$.

Consider also the three discernibility systems $(S,\mathcal {D},\mathcal {T}_1,\delta _1)$, $(S,\mathcal {D},\mathcal {T}_2,\delta _2)$, and $(S,\mathcal {D},\mathcal {T}_3,\delta _3)$, where *S* and $\mathcal {D}$ are defined as above, and the competency set $\mathcal {T}$ is restricted to the subsets $\mathcal {T}_1 = \{a,ab,cd\}$, $\mathcal {T}_2 = \{c,ab,acd\}$, $\mathcal {T}_3 = \{ab,bd,acd\}$ with the respective discernibility functions $\delta _1, \delta _2$, and $\delta _3$ as specified in Table 1. $(S,\mathcal {D},\mathcal {T}_1,\delta _1)$ is a reduct of $(S,\mathcal {D},\mathcal {T},\delta )$ because none of the three competencies in $\mathcal {T}_1$ can be removed without emptying some bag: $\delta _1(ac,bd)=\{a\}$, $\delta _1(ab,ad)=\{ab\}$, $\delta _1(bd,bcd)=\{cd\}$. $(S,\mathcal {D},\mathcal {T}_2,\delta _2)$ is a simplification of $(S,\mathcal {D},\mathcal {T},\delta )$ but not a reduct because competency *acd* can be removed from $\mathcal {T}_2$ without emptying any bag: for $\{acd\}\in \mathcal {T}_2$, there is no pair $\{C,C'\}\in \mathcal {D}$ such that $\delta _2(C,C')=\{acd\}$. $(S,\mathcal {D},\mathcal {T}_3,\delta _3)$ is not simplification of $(S,\mathcal {D},\mathcal {T},\delta )$ because pair $\{bd,bcd\}$ is distinguished by the competencies in $\mathcal {T}$ but not by those in $\mathcal {T}_3$: $\delta (bd,bcd) = \{c,cd,bcd\} \ne \emptyset$ whereas $\delta _3(bd,bcd) = \emptyset$.

Reducts play a fundamental role in developing tests that are as informative as possible and minimal, in the sense that eliminating any of their items would make them less informative. Consider a reduct $(S,\mathcal {D},\mathcal {T}^\diamond ,\delta ^\diamond )$ of $(S,\mathcal {D},\mathcal {T},\delta )$. To construct a conjunctive competence model based on this reduct, a set *Q* of items must be defined such that, for every $T \in \mathcal {T}^\diamond$, there is some item q∈Q such that $\mu (q)=\{T\}$. This yields a competence model $(Q,S,\mathcal {C},\mu )$ that is as informative as possible given the competencies in $\mathcal {T}$ (i.e., a competence model that is as informative as a hypothetical conjunctive competence model containing some item for every competency in $\mathcal {T}$). Furthermore, if *Q* contains only one item for each $T \in \mathcal {T}^\diamond$, $(Q,S,\mathcal {C},\mu )$ is also minimal. There may exist various distinct reducts of a discernibility system. For instance, a reduct of the discernibility system $(S,\mathcal {D},\mathcal {T},\delta )$ in Example 3 has competency set $\mathcal {T}_1=\{a,ab,cd\}$. Another one has competency set $\{c,ab\}$. Tests based on distinct reducts of the same discernibility system differ in items and associated competencies, but are equally informative about the competence states underlying the knowledge states.

**Table 2:** Run of the competency deletion procedure in Example 5, with random selection of competencies for deletion

*k*	$\mathbb {M}_k$	$\mathcal {E}_k$
0	$\{\{b\},\{a,ab\},\{a,c,ac\}$,$\{c,ac,bc,abc\},\{c,bc,bd,cd,bcd\}$,$\{ad,bd,cd,abd,acd,bcd,abcd\}\}$	$\{a,\boldsymbol{c},ab,ac,ad,bc,bd,cd,abc,abd,acd,bcd,abcd\}$
1	$\{\{b\},\{a,ab\},\{a,ac\},\{ac,bc,abc\}$,$\{bc,bd,cd,bcd\},\{ad,bd,cd,abd,acd,bcd,abcd\}\}$	$\{a,ab,ac,ad,\boldsymbol{bc},bd,cd,abc,abd,acd,bcd,abcd\}$
2	$\{\{b\},\{a,ab\},\{a,ac\},\{ac,abc\},\{bd,cd,bcd\}\}$	$\{\boldsymbol{a},ab,ac,bd,cd,abc,bcd\}$
3	$\{\{b\},\{ab\},\{ac\},\{bd,cd,bcd\}\}$	$\{bd,cd,\boldsymbol{bcd}\}$
4	$\{\{b\},\{ab\},\{ac\},\{bc,cd\}\}$	$\{\boldsymbol{bc},cd\}$
5	$\{\{b\},\{ab\},\{ac\},\{cd\}\}$	∅

*Note.* Selected competencies are in bold

### Competency deletion procedure

The CDP constructs a reduct of the discernibility system $(S,\mathcal {D},\mathcal {T},\delta )$ by iteratively removing competencies from the full set $\mathcal {T}$. The procedure in Anselmi et al. (2022) removes one competency from $\mathcal {T}$ per iteration, whereas the more efficient variant in Anselmi et al. (2024b) removes multiple competencies per iteration. This section describes the latter, called the set-based CDP in Anselmi et al. (2024b) and referred to here simply as CDP. The notions of bag set and minimal bag set are relevant in the construction that follows. In particular, the *bag set* of the discernibility system $(S,\mathcal {D},\mathcal {T},\delta )$ is the collection $\mathbb {B}$ of all nonempty bags of $(S,\mathcal {D},\mathcal {T},\delta )$:

2 $$\begin{aligned} \mathbb {B}= \{\delta (C,C') : \delta (C,C') \ne \emptyset , \{C,C'\} \in \mathcal {D}\}. \end{aligned}$$

Denoting by $\mathcal {B}$ an arbitrary bag in $\mathbb {B}$, the *minimal bag set* of $\mathbb {B}$ is the collection $\mathbb {M}$ of all bags $\mathcal {B}$ in $\mathbb {B}$ that are minimal with respect to set inclusion, that is, that do not properly include any other bag in $\mathbb {B}$:

3 $$\begin{aligned} \mathbb {M}= \{\mathcal {B}\in \mathbb {B}: \mathcal {B}\not \supset \mathcal {B}' \text { for all } \mathcal {B}' \in \mathbb {B}\}. \end{aligned}$$

The construction of $\mathbb {M}$ is justified by the fact that the competencies in minimal bags are sufficient to distinguish the same pairs of competence states that can be distinguished by the competencies in the complete bag set $\mathbb {B}$.

#### Example 4

The bag set of discernibility system $(S,\mathcal {D},\mathcal {T},\delta )$ in Example 3 is the collection $\mathbb {B}= \{\{ab\},\{a,c,bd\},\{c,cd,bcd\},\{ab,cd,acd\}\}$ and the minimal bag set is the collection $\mathbb {M}= \{\{ab\},\{a,c,bd\},\{c,cd,bcd\}\}$. Bag $\{ab,cd,acd\}$ in $\mathbb {B}$ does not belong to $\mathbb {M}$ because it properly includes bag $\{ab\}$.

Let $(S,\mathcal {D},\mathcal {T},\delta )$ be the discernibility system of which a reduct is to be constructed, and whose bag set $\mathbb {B}$ is obtained by an application of Eq. 2. The scalar *k* denotes the iteration of the procedure and $\mathbb {B}_k$ the bag set at *k*. The initial bag set is set to be $\mathbb {B}_0= \mathbb {B}$. At each iteration *k*, the procedure performs the following steps:

**1**: **Identification of the minimal bag set.** Obtain the minimal bag set $\mathbb {M}_k$ from the bag set $\mathbb {B}_k$ by an application of Eq. 3.
**2**: **Identification of the competencies that can be deleted.** Define $\mathcal {E}_k = \bigcup \{\mathcal {B}\in \mathbb {M}_k: |\mathcal {B}| > 1\}$ as the set of all competencies contained in the nonsingleton bags in $\mathbb {M}_k$, that is, all competencies that can be deleted at *k* (|*X*| denotes the cardinality of set *X*).
**3**: **Evaluation of the stopping criterion.** Stop at *k* if there is no competency that can be deleted, that is, if $\mathcal {E}_k$ is empty.
**4**: **Selection of one competency for deletion.** If the stopping criterion is not satisfied at *k*, select from $\mathcal {E}_k$ the competency *E* to be deleted. Define $\mathbb {B}_{k+1} = \{\mathcal {B}\setminus \{E\}: \mathcal {B}\in \mathbb {M}_k\}$ as the bag set at k+1, that is, the set of bags obtained by removing *E* from each bag in $\mathbb {M}_k$.

Let $\mathcal {T}_{\mathbb {B}_k} = \bigcup \mathbb {B}_k$ denote the set of all competencies contained in the bags of $\mathbb {B}_k$. Since $\mathbb {B}_{k+1}$ is derived from $\mathbb {M}_k$, $\mathcal {T}_{\mathbb {B}_{k+1}} = \bigcup \mathbb {B}_{k+1}$ lacks both the competency $E \in \mathcal {E}_k$ deleted at *k* and all competencies not included in minimal bags at *k*. The procedure cycles through steps 1 to 4 until the stopping criterion is satisfied. Assuming that it stops at iteration k=l, the reduct $(S,\mathcal {D},\mathcal {T}^\diamond ,\delta ^\diamond )$ of $(S,\mathcal {D},\mathcal {T},\delta )$ is obtained, where $\mathcal {T}^\diamond = \bigcup \mathbb {M}_l$.

Distinct reducts of a discernibility system $$(S,\mathcal {D},\mathcal {T},\delta )$$ can be obtained by deleting one competency in place of another when there is a choice (i.e., when $$|\mathcal {E}_k|>1$$ for some k≥0). The competency *E* to be deleted at iteration *k* can be selected from $$\mathcal {E}_k$$ randomly or based on a preference relation ≿ defined on the competencies in $$\mathcal {T}$$. For any two competencies $$T,T' \in \mathcal {T}$$, $T≿T^{\prime }$ means that *T* is weakly preferred to $T^{\prime }$. The relation ≿ is assumed to be a weak order (transitive, reflexive, and connected relation), and it includes the indifference relation ∼ defined by $T∼T^{\prime }$ if $T≿T^{\prime }$ and $T^{\prime }≿T$, which is an equivalence relation, and the (strict) preference relation ≻ defined by $T≻T^{\prime }$ if $$T' \not \succsim T$$, which is a strict weak order (negatively transitive and asymmetric relation). For any two competencies $$T,T' \in \mathcal {T}$$, $T∼T^{\prime }$ means that *T* is as preferred as $T^{\prime }$, and $T≻T^{\prime }$ means that *T* is strictly preferred to $T^{\prime }$. The *delete least preferred rule* (Anselmi et al., 2024b) prescribes selecting any competency in $$\mathcal {E}_k$$ that is least preferred (i.e., rightmost) based on ≿. If more than one competency in $$\mathcal {E}_k$$ satisfies this criterion, one of them is chosen at random. The random selection of *E* from $$\mathcal {E}_k$$ yields any possible reduct of the discernibility system. The selection of *E* based on ≿ produces those reducts most preferred under the right-to-left lexical order $$\gg _\text {R2L}$$ induced by ≿ on the competency sets of the reducts (for details see Anselmi et al., 2024b). Specifically, those reducts contain the lowest number of competencies that are least preferred, second least preferred, and so on, according to ≿.

#### Example 5

Given the set of skills $S = \{a,b,c,d\}$ and the competence structure $\mathcal {C} = \{\emptyset ,b,ab,ac,abc,bcd,abcd\}$ in Example 1, consider the discernibility system $(S,\mathcal {D},\mathcal {T},\delta )$ with  and

$$ \mathcal {T}= \{a, b, c, ab, ac, ad, bc, bd, cd, abc, abd, acd, bcd, abcd\}. $$

With $\mathbb {B}$ being the bag set of $(S,\mathcal {D},\mathcal {T},\delta )$, the initial bag set is assumed to be $\mathbb {B}_0 = \mathbb {B}$. Table 2 shows a possible run of the CDP with random selection of the competency for deletion. The minimal bag set $\mathbb {M}_k$ and the collection of deletable competencies $\mathcal {E}_k$ are shown, while the bag set $\mathbb {B}_k$ is omitted for reasons of space. Suppose the bolded competency in $\mathcal {E}_k$ is randomly selected and deleted at iteration *k*. The procedure stops at k=5 (where $\mathcal {E}_5 = \emptyset$) and leads to the reduct $(S,\mathcal {D},\mathcal {T}^\diamond ,\delta ^\diamond )$ of $(S,\mathcal {D},\mathcal {T},\delta )$, with competency set $\mathcal {T}^\diamond =\bigcup \mathbb {M}_5=\{b,ab,ac,cd\}$. A new run of the CDP with random selection of the competency for deletion may yield any of the 34 reducts of $(S,\mathcal {D},\mathcal {T},\delta )$, whose competency sets are listed in Table 3.

**Table 3:** Competency sets $\mathcal {T}^\diamond _j$ of the 34 reducts $(S,\mathcal {D},\mathcal {T}^\diamond _j,\delta ^\diamond _j)$, j=1,...,34, of the discernibility system in Example 5 and iteration *k* at which the competency deletion procedure (CDP) and the competency addition procedure (CAP) satisfied the stopping criterion

			Iteration *k*						Iteration *k*	
*j*	$\mathcal {T}^\diamond _j$	$|\mathcal {T}^\diamond _j|$	CDP	CAP		*j*	$\mathcal {T}^\diamond _j$	$|\mathcal {T}^\diamond _j|$	CDP	CAP
1	$\{b,c,ab,abd\}$	4	8-10	2-3		18	$\{b,c,ab,acd\}$	4	8-10	2-3
2	$\{b,ab,ac,cd\}$	4	5-10	2-3		19	$\{a,b,bc,cd\}$	4	9-10	2-3
3	$\{b,c,ab,ad\}$	4	8-10	2-3		20	$\{a,b,abc,bcd\}$	4	5-10	2-3
4	$\{a,b,ac,bd\}$	4	6-10	3		21	$\{b,c,ab,bcd\}$	4	8-10	2-3
5	$\{b,ab,ac,bc,abd\}$	5	8-9	2-3		22	$\{b,c,ab,abcd\}$	4	8-10	2-3
6	$\{a,b,cd,abc\}$	4	5-10	2-3		23	$\{a,b,bc,acd\}$	4	8-10	2-3
7	$\{b,ab,ac,bd\}$	4	5-10	2-3		24	$\{a,b,bc,bd\}$	4	9-10	2-3
8	$\{a,b,c,bcd\}$	4	10	3		25	$\{b,c,ab,cd\}$	4	8-10	2-3
9	$\{a,b,ad,bc\}$	4	8-10	2-3		26	$\{a,b,c,cd\}$	4	10	3
10	$\{b,c,ab,bd\}$	4	8-10	2-3		27	$\{a,b,c,abcd\}$	4	8-10	3
11	$\{a,b,ac,bcd\}$	4	6-10	3		28	$\{a,b,c,bd\}$	4	10	3
12	$\{b,ab,ac,bcd\}$	4	5-10	2-3		29	$\{a,b,bc,abd\}$	4	8-10	2-3
13	$\{a,b,bd,abc\}$	4	5-10	2-3		30	$\{b,ab,ac,bc,acd\}$	5	8-9	2-3
14	$\{a,b,ac,cd\}$	4	6-10	3		31	$\{a,b,bc,abcd\}$	4	8-10	2-3
15	$\{a,b,bc,bcd\}$	4	9-10	2-3		32	$\{b,ab,ac,bc,abcd\}$	5	8-9	2-3
16	$\{a,b,c,abd\}$	4	8-10	3		33	$\{a,b,c,acd\}$	4	8-10	3
17	$\{a,b,c,ad\}$	4	8-10	3		34	$\{b,ab,ac,ad,bc\}$	5	8-9	2-4

*Note.* $|\mathcal {T}^\diamond _j|$: cardinality of $\mathcal {T}^\diamond _{j}$

**Table 4:** Run of the competency deletion procedure in Example 5, with selection of competencies for deletion based on the preference relation in Eq. 4

*k*	$\mathbb {M}_k$	$\mathcal {E}_k$
0	$\{\{b\},\{a,ab\},\{a,c,ac\}$, $\{c,ac,bc,abc\},\{c,bc,bd,cd,bcd\}$, $\{ad,bd,cd,abd,acd,bcd,abcd\}\}$	$\{a,c,\underline{ab},\underline{ac},ad,bc,\underline{bd},cd,abc,\underline{\boldsymbol{abd}},acd,bcd,abcd\}$
1	$\{\{b\},\{a,ab\},\{a,c,ac\},\{c,ac,bc,abc\}$, $\{c,bc,bd,cd,bcd\},\{ad,bd,cd,acd,bcd,abcd\}\}$	$\{a,c,\underline{ab},\underline{ac},ad,bc,\underline{\boldsymbol{bd}},cd,abc,acd,bcd,abcd\}$
2	$\{\{b\},\{a,ab\},\{a,c,ac\},\{c,ac,bc,abc\}$, $\{c,bc,cd,bcd\},\{ad,cd,acd,bcd,abcd\}\}$	$\{a,c,\underline{ab},\underline{\boldsymbol{ac}},ad,bc,cd,abc,acd,bcd,abcd\}$
3	$\{\{b\},\{a,ab\},\{a,c\},\{c,bc,abc\},\{c,bc,cd,bcd\}$, $\{ad,cd,acd,bcd,abcd\}\}$	$\{a,c,\underline{\boldsymbol{ab}},ad,bc,cd,abc,acd,bcd,abcd\}$
4	$\{\{b\},\{a\},\{c,bc,abc\},\{c,bc,cd,bcd\}$, $\{ad,cd,acd,bcd,abcd\}\}$	$\{c,ad,bc,cd,abc,\underline{\boldsymbol{acd}},bcd,abcd\}$
5	$\{\{b\},\{a\},\{c,bc,abc\},\{c,bc,cd,bcd\}$, $\{ad,cd,bcd,abcd\}\}$	$\{\underline{c},ad,\underline{bc},\underline{\boldsymbol{cd}},abc,\underline{bcd},\underline{abcd}\}$
6	$\{\{b\},\{a\},\{c,bc,abc\},\{c,bc,bcd\}$, $\{ad,bcd,abcd\}\}$	$\{\underline{\boldsymbol{c}},ad,\underline{bc},abc,\underline{bcd},\underline{abcd}\}$
7	$\{\{b\},\{a\},\{bc,abc\},\{bc,bcd\},\{ad,bcd,abcd\}\}$	$\{ad,\underline{\boldsymbol{bc}},abc,\underline{bcd},\underline{abcd}\}$
8	$\{\{b\},\{a\},\{abc\},\{bcd\}\}$	∅

*Note.* Least preferred competencies are underlined, and selected competencies are in bold

Consider the preference relation ≿ on $\mathcal {T}$ defined as

4 $$\begin{aligned} &  ad \sim abc \succ c \sim bc \sim cd \sim bcd \sim abcd \succ a \sim b \sim acd\nonumber \\ &  \qquad \succ ab \sim ac \sim bd \sim abd, \end{aligned}$$

according to which, for instance, competencies *ad* and *abc* are indifferent and preferred to competencies *bc* and *acd*. Table 4 illustrates a possible run of the CDP based on this preference relation. Suppose that, at each iteration *k*, the bolded competency in $\mathcal {E}_k$ is randomly selected from the least preferred competencies in Eq. 4 (which are underlined) and deleted. Then, the procedure stops at k=5 (where $\mathcal {E}_5 = \emptyset$) and leads to the reduct $(S,\mathcal {D},\mathcal {T}^\diamond ,\delta ^\diamond )$ of $(S,\mathcal {D},\mathcal {T},\delta )$, with competency set $\mathcal {T}^\diamond = \bigcup \mathbb {M}_5 = \{a,b,abc,bcd\}$. This is one of the four $\gg _\text {R2L}$-most-preferred reducts of $(S,\mathcal {D},\mathcal {T},\delta )$, namely, the one with competency set $\mathcal {T}^\diamond _{20}$ (see Table 3). The other three have competency sets $\mathcal {T}^\diamond _6$, $\mathcal {T}^\diamond _9$, and $\mathcal {T}^\diamond _{17}$. Each of these four reducts contains two second-least-preferred competencies, one third-least-preferred competency, and one fourth-least-preferred competency, whereas the remaining 30 reducts contain more second-, third-, or fourth-least-preferred competencies. A new run of the CDP based on Eq. 4 may yield any of these four reducts.

## Partial reduct and core

This section introduces some new concepts that form the basis of the CAP in Section “Competency addition procedure”. Both the concepts and the CAP were inspired by RST, particularly by the work of Yao and Zhao (2009), and Yao et al. (2006); Yao, Zhao and Wang (2008).

### Definition 1

(Partial reduct). A *partial reduct* of the discernibility system $(S,\mathcal {D},\mathcal {T},\delta )$ is a quadruple $(S,\mathcal {D},\mathcal {T}^\bullet ,\delta ^\bullet )$ for which there exists a reduct $(S,\mathcal {D},\mathcal {T}^\diamond ,\delta ^\diamond )$ of $(S,\mathcal {D},\mathcal {T},\delta )$ with $\mathcal {T}^\bullet \subset \mathcal {T}^\diamond$ and $\delta ^\bullet (C,C')=\delta ^\diamond (C,C') \cap \mathcal {T}^\bullet$ for each pair $\{C,C'\}\in \mathcal {D}$.

According to Definition 1, given a reduct $(S,\mathcal {D},\mathcal {T}^\diamond ,\delta ^\diamond )$ of the discernibility system $(S,\mathcal {D},\mathcal {T},\delta )$, there can be more than one partial reduct of $(S,\mathcal {D},\mathcal {T},\delta )$. Each competency in a partial reduct is necessary to distinguish some pairs of competence states distinguished by the original discernibility system, but together they are not sufficient to distinguish all the pairs distinguished by it.

### Example 6

Consider the reduct $(S,\mathcal {D},\mathcal {T}^\diamond _1, \delta ^\diamond _1)$ of the discernibility system $(S,\mathcal {D},\mathcal {T},\delta )$ in Example 5, with $\mathcal {T}^\diamond _1 = \{b,c,ab,abd\}$ (see Table 3). Each quadruple $(S,\mathcal {D},\mathcal {T}^\bullet ,\delta ^\bullet )$ with $\mathcal {T}^\bullet \subset \mathcal {T}^\diamond _1$ is a partial reduct of $(S,\mathcal {D},\mathcal {T},\delta )$. For instance, $(S,\mathcal {D},\mathcal {T}^\bullet ,\delta ^\bullet )$ with $\mathcal {T}^\bullet = \{b,c,abd\}$ is one such partial reduct.

Because a reduct $(S,\mathcal {D},\mathcal {T}^\diamond ,\delta ^\diamond )$ of a discernibility system $(S,\mathcal {D},\mathcal {T},\delta )$ is a minimal simplification of it, we immediately have the following result.

**Fig. 1:**
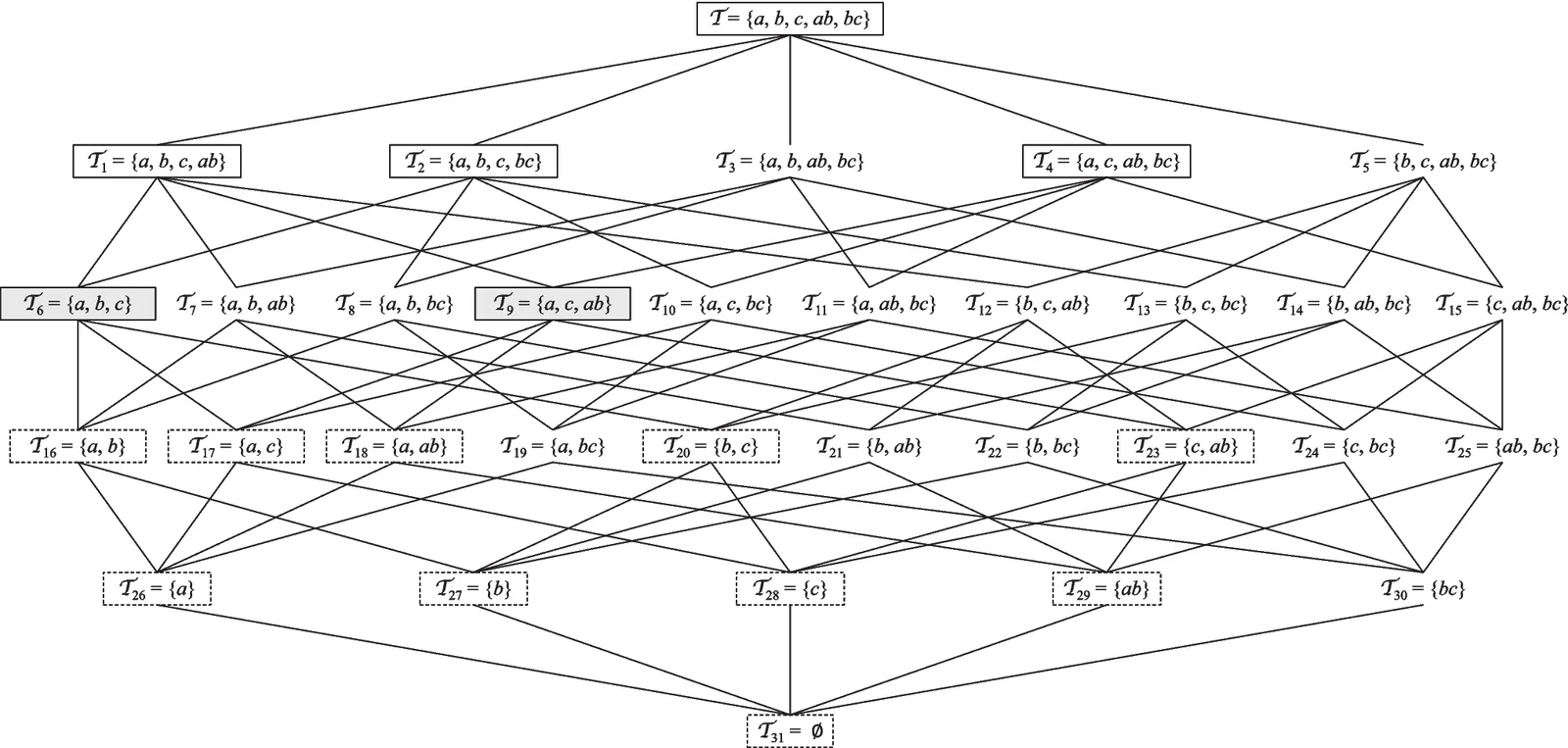
Subsets of $\mathcal {T}= \{a,b,c,ab,bc\}$, where those defining simplifications of the discernibility system $(S,\mathcal {D},\mathcal {T},\delta )$ in Example 8 are in solid-line rectangles, those defining reducts are in shaded rectangles, and those defining partial reducts are in dashed-line rectangles

### Proposition 1

Any partial reduct $(S,\mathcal {D},\mathcal {T}^\bullet ,\delta ^\bullet )$ of a discernibility system $(S,\mathcal {D},\mathcal {T},\delta )$ is not a simplification of $(S,\mathcal {D},\mathcal {T},\delta )$.

### Definition 2

(Core). Consider the bag set $\mathbb {B}$ of a discernibility system $(S,\mathcal {D},\mathcal {T},\delta )$. The *core* of $(S,\mathcal {D},\mathcal {T},\delta )$ is the collection $\mathcal {T}^c \subseteq \mathcal {T}$ of all competencies that occur as the sole element of some bag in $\mathbb {B}$:

5 $$\begin{aligned} \mathcal {T}^c = \{T \in \mathcal {T}: \{T\}\in \mathbb {B}\}. \end{aligned}$$

Obviously, if $\mathbb {M}$ is the minimal bag set of $\mathbb {B}$, then $\mathcal {T}^c = \{T \in \mathcal {T}: \{T\}\in \mathbb {M}\}$. Each competency in $\mathcal {T}^c$ is a *core competency* and, since it is the only competency in $\mathcal {T}$ capable of distinguishing some pair $\{C,C'\} \in \mathcal {D}$, it is necessarily contained in any reduct of $(S,\mathcal {D},\mathcal {T},\delta )$. This in turn means that any discernibility system whose competency set does not include all core competencies in $\mathcal {T}^c$ cannot be a reduct of $(S,\mathcal {D},\mathcal {T},\delta )$. Moreover, any discernibility system whose competency set is a proper subset of $\mathcal {T}^c$ is a partial reduct of $(S,\mathcal {D},\mathcal {T},\delta )$. The following result provides an alternative characterization of the core of a discernibility system, which is shown to be equivalent to Definition 2.

### Proposition 2

Consider the collection of all reducts $(S,\mathcal {D},\mathcal {T}^\diamond _j,\delta ^\diamond _j)$, j=1,...,J, of the discernibility system $(S,\mathcal {D},\mathcal {T},\delta )$. The core $\mathcal {T}^c$ of $(S,\mathcal {D},\mathcal {T},\delta )$ as defined by Eq. 5 is equal to $\bigcap _{j=1}^J \mathcal {T}^\diamond _j$.

### Proof

$\mathcal {T}^c \subseteq \bigcap _{j=1}^J \mathcal {T}^\diamond _j$: Since each competency $T \in \mathcal {T}^c$ is necessary to distinguish some pair $\{ C , C' \}\in \mathcal {D}$ (i.e., $\delta (C,C')=\{T\}$), it must belong to each of the collections $\mathcal {T}^\diamond _j$, j=1,...,J, and therefore lies in $\bigcap _{j=1}^J \mathcal {T}^\diamond _j$.

$\bigcap _{j=1}^J \mathcal {T}^\diamond _j \subseteq \mathcal {T}^c$: Assume that $\bigcap _{j=1}^J \mathcal {T}^\diamond _j \not \subseteq \mathcal {T}^c$. This implies that all bags containing a competency $T \in \bigcap _{j=1}^J \mathcal {T}^\diamond _j \setminus \mathcal {T}^c$ also contain at least one other competency besides *T*. But then $(S,\mathcal {D},\mathcal {T}^\diamond ,\delta ^\diamond )$, with $\mathcal {T}^\diamond = \mathcal {T}\setminus \{T\}$ is a simplification of $(S,\mathcal {D},\mathcal {T},\delta )$, which in turn means that there is some reduct $(S,\mathcal {D},\mathcal {T}^\diamond _j,\delta ^\diamond _j)$ of $(S,\mathcal {D},\mathcal {T},\delta )$ such that $T \not \in \mathcal {T}^\diamond _j$. So, it follows that $T \not \in \bigcap _{j=1}^J \mathcal {T}^\diamond _j$, a contradiction.□

The core $\mathcal {T}^c$ of a discernibility system $(S,\mathcal {D},\mathcal {T},\delta )$ is the subset of $\mathcal {T}$ that contains all the competencies necessary to construct every possible reduct of $(S,\mathcal {D},\mathcal {T},\delta )$. Given a reduct $(S,\mathcal {D},\mathcal {T}^\diamond ,\delta ^\diamond )$ of $(S,\mathcal {D},\mathcal {T},\delta )$, any proper superset of $\mathcal {T}^c$ that is a subset of $\mathcal {T}^\diamond$ allows for obtaining only some of the reducts of $(S,\mathcal {D},\mathcal {T},\delta )$.

### Example 7

Consider the discernibility system $(S,\mathcal {D},\mathcal {T},\delta )$ in Example 5. With $\mathbb {M}_0$ in Table 2 corresponding to the minimal bag set of the bag set $\mathbb {B}$ of $(S,\mathcal {D},\mathcal {T},\delta )$, it is clear that the core of $(S,\mathcal {D},\mathcal {T},\delta )$ is $\mathcal {T}^c = \{b\}$. Given the set of the 34 reducts $(S,\mathcal {D},\mathcal {T}^\diamond _j,\delta ^\diamond _j)$, j=1,...,34, of $(S,\mathcal {D},\mathcal {T},\delta )$, whose competency sets are listed in Table 3, it is easy to see that $\bigcap _{j=1}^{34} \mathcal {T}^\diamond _j = \{b\} = \mathcal {T}^c$. Given the reduct with competency set $\mathcal {T}^\diamond _1 = \{b,c,ab,abd\}$, the collection $\{b,ab,abd\}$, with $\mathcal {T}^c \subset \{b,ab,abd\} \subseteq \mathcal {T}^\diamond _1$, allows for obtaining two of the 34 reducts of $(S,\mathcal {D},\mathcal {T},\delta )$, namely those with competency sets $\mathcal {T}^\diamond _1$ and $\mathcal {T}^\diamond _5$.

The following immediate results provide useful insights into how simplifications, reducts, partial reducts, and the core of a discernibility system relate to one another. Given any three discernibility systems $(S,\mathcal {D},\mathcal {T},\delta )$, $(S,\mathcal {D},\mathcal {T}_1,\delta _1)$, and $(S,\mathcal {D},\mathcal {T}_2,\delta _2)$, with $\mathcal {T}_1,\mathcal {T}_2 \subseteq \mathcal {T}$ and $\mathcal {T}_1 \ne \mathcal {T}_2$:

- If $(S,\mathcal {D},\mathcal {T}_1,\delta _1)$ and $(S,\mathcal {D},\mathcal {T}_2,\delta _2)$ are two reducts of $(S,\mathcal {D},\mathcal {T},\delta )$, then the discernibility system based on $\mathcal {T}_1 \cup \mathcal {T}_2$ is a simplification but not a reduct of $(S,\mathcal {D},\mathcal {T},\delta )$.
- If $(S,\mathcal {D},\mathcal {T}_1,\delta _1)$ and $(S,\mathcal {D},\mathcal {T}_2,\delta _2)$ are two reducts of $(S,\mathcal {D},\mathcal {T},\delta )$, then the discernibility system based on $\mathcal {T}_1 \cap \mathcal {T}_2$ is a partial reduct of $(S,\mathcal {D},\mathcal {T},\delta )$.
- If $(S,\mathcal {D},\mathcal {T}_1,\delta _1)$ and $(S,\mathcal {D},\mathcal {T}_2,\delta _2)$ are two simplifications of $(S,\mathcal {D},\mathcal {T},\delta )$, then the discernibility system based on $\mathcal {T}_1 \cap \mathcal {T}_2$ does not need to be a partial reduct of $(S,\mathcal {D},\mathcal {T},\delta )$.

Moreover, given the core $\mathcal {T}^c$ of $(S,\mathcal {D},\mathcal {T},\delta )$, defined according to Eq. 5:

- $(S,\mathcal {D},\mathcal {T}^c,\delta ^c)$ is either a reduct or a partial reduct of $(S,\mathcal {D},\mathcal {T},\delta )$.
- $(S,\mathcal {D},\mathcal {T}^c,\delta ^c)$ is a reduct of $(S,\mathcal {D},\mathcal {T},\delta )$ if and only if it is the only reduct of $(S,\mathcal {D},\mathcal {T},\delta )$.

### Example 8

Given the set of skills $S = \{a,b,c\}$ and the competence structure $\mathcal {C} = \{\emptyset ,a,ab,ac,abc\}$, consider the discernibility system $(S,\mathcal {D},\mathcal {T},\delta )$ with  and $\mathcal {T}= \{a,b,c,ab,bc\}$. Figure 1 shows all subsets of $\mathcal {T}$. The subsets defining simplifications of $(S,\mathcal {D},\mathcal {T},\delta )$ are in solid-line rectangles, those defining reducts are in shaded rectangles, and those defining partial reducts are in dashed-line rectangles. $\mathcal {T}_6=\{a,b,c\}$ and $\mathcal {T}_9=\{a,c,ab\}$ define reducts of $(S,\mathcal {D},\mathcal {T},\delta )$, $\mathcal {T}_6\cup \mathcal {T}_9=\mathcal {T}_1=\{a,b,c,ab\}$ defines a simplification but not a reduct, and $\mathcal {T}_6\cap \mathcal {T}_9=\mathcal {T}_{17}=\{a,c\}$ defines a partial reduct. $\mathcal {T}_2=\{a,b,c,bc\}$ and $\mathcal {T}_4=\{a,c,ab,bc\}$ define simplifications of $(S,\mathcal {D},\mathcal {T},\delta )$, but $\mathcal {T}_2\cap \mathcal {T}_4=\mathcal {T}_{10}=\{a,c,bc\}$ does not define a partial reduct: $\mathcal {T}_{10}$ is a subset of neither $\mathcal {T}_6=\{a,b,c\}$ nor $\mathcal {T}_9=\{a,c,ab\}$, the competency sets defining the two reducts. The core $\mathcal {T}^c=\mathcal {T}_{17}=\{a,c\}$ of $(S,\mathcal {D},\mathcal {T},\delta )$ defines a partial reduct.

**Table 5:** Run of the competency addition procedure in Example 9, with random selection of competencies for addition and bags

*k*	$\mathbb {M}_k$	$\mathcal {A}_k$
0	$\{\{b\},\{a,ab\},\{a,c,ac\}$,$\{c,ac,bc,abc\}$, $\underline{\boldsymbol{\{c,bc,bd,cd,bcd\}}}$,$\underline{\{ad,bd,cd,abd,acd,bcd,abcd\}}\}$	$\{a,c,ab,ac,ad,bc,bd,\boldsymbol{cd},abc,abd,acd,bcd,abcd\}$
1	$\{\{b\},\underline{\boldsymbol{\{a,ab\}}},\{a,ac\},\{ac,abc\},\{cd\}\}$	$\{a,\boldsymbol{ab},ac,abc\}$
2	$\{\{b\},\{ab\},\{ac\},\{cd\}\}$	∅

*Note.* Selected competencies are in bold. Bags containing selected competencies are underlined, and selected bags are in bold

## Competency addition procedure

The CAP constructs a reduct of $(S,\mathcal {D},\mathcal {T},\delta )$ by iteratively adding competencies to the core $\mathcal {T}^c$ of $(S,\mathcal {D},\mathcal {T},\delta )$. Let $(S,\mathcal {D},\mathcal {T},\delta )$ be the discernibility system of which a reduct is to be constructed, and whose bag set $\mathbb {B}$ is obtained by an application of Eq. 2. With *k* denoting the iteration of the procedure, let $\mathbb {B}_k$ be the bag set at *k*. The initial bag set at k=0 is set to be $\mathbb {B}_0 = \mathbb {B}$. At each iteration *k*, the procedure performs the following steps:

**1**: **Identification of the minimal bag set.** Obtain the minimal bag set $\mathbb {M}_k$ from the bag set $\mathbb {B}_k$ by an application of Eq. 3.
**2**: **Identification of the competencies that can be added.** Define $\mathcal {A}_k = \bigcup \{\mathcal {B}\in \mathbb {M}_k:|\mathcal {B}|>1\}$ as the set of all competencies contained in the nonsingleton bags in $\mathbb {M}_k$, that is, all competencies that can be added at *k*.
**3**: **Evaluation of the stopping criterion.** Stop at *k* if there is no competency that can be added, that is, if $\mathcal {A}_k$ is empty.
**4**: **Selection of one competency for addition.** If the stopping criterion is not satisfied at *k*, select from $\mathcal {A}_k$ the competency *A* to be added and, from the set $\mathbb {M}^A_k = \{\mathcal {B}\in \mathbb {M}_k:A \in \mathcal {B}\}$ of all bags in $\mathbb {M}_k$ containing *A*, a bag $\mathcal {B}^A$. Define $\mathbb {B}_{k+1} = \{\mathcal {B}\setminus (\mathcal {B}^A\setminus \{A\}):\mathcal {B}\in \mathbb {M}_k\}$ as the bag set at k+1, that is, the set of bags obtained by removing every competency in $\mathcal {B}^A$ other than *A* from each bag in $\mathbb {M}_k$.

At each iteration *k*, we consider the discernibility system $(S,\mathcal {D},\mathcal {T}_k,\delta _k)$, where $\mathcal {T}_k = \bigcup \{\mathcal {B}\in \mathbb {M}_k:|\mathcal {B}| = 1\}$ is the collection of all competencies that are necessary to distinguish some pair in $\mathcal {D}$, because each is the sole element of some bag in $\mathbb {M}_k$. The following proposition shows that the procedure stops if and only if a reduct of $(S,\mathcal {D},\mathcal {T},\delta )$ is obtained.

### Proposition 3

The termination criterion is satisfied at iteration k=l if and only if the discernibility system $(S,\mathcal {D},\mathcal {T}_{l},\delta _{l})$, with $\mathcal {T}_{l} = \bigcup \mathbb {M}_{l} = \bigcup \{\mathcal {B}\in \mathbb {M}_l:|\mathcal {B}| = 1\}$, is a reduct of $(S,\mathcal {D},\mathcal {T},\delta )$.

### Proof

Let $\mathcal {D}^\circ \subseteq \mathcal {D}$ be the subset of pairs of competence states distinguished by $(S,\mathcal {D},\mathcal {T},\delta )$; that is, $\mathcal {D}^\circ = \{\{C,C'\}\in \mathcal {D}: \delta (C,C')\ne \emptyset \}$. At each iteration *k* the set $\mathcal {D}^\circ$ is partitioned into $\mathcal {D}^+_k$ by the collection $\mathcal {T}_k$ of competencies that, at *k*, are necessary to distinguish some pair in $\mathcal {D}^\circ$, and the pairs of competence states $\mathcal {D}^-_k = \mathcal {D}^\circ \setminus \mathcal {D}^+_k$ distinguished by some competency in $\mathcal {A}_k$. Performing the step from *k* to k+1 transfers at least one pair $\{C, C'\} \in \mathcal {D}^-_k$ to $\mathcal {D}^+_{k+1}$. This continues until k=l, where $\mathcal {A}_l = \emptyset$, which in turn holds if and only if $\mathcal {D}^-_l = \emptyset$. This means that $\mathcal {D}^+_l = \mathcal {D}^\circ$ and thus $(S,\mathcal {D},\mathcal {T}_{l},\delta _{l})$ is a simplification of $(S,\mathcal {D},\mathcal {T},\delta )$.

It remains to show that $(S,\mathcal {D},\mathcal {T}_{l},\delta _{l})$ is in fact a reduct, that is, we cannot delete any $T \in \mathcal {T}_l$ without losing the property of distinguishing all pairs in $\mathcal {D}^\circ$. It is clear from Proposition 2 that we cannot delete any competency from the core $\mathcal {T}_0 = \mathcal {T}^c$ of $(S,\mathcal {D},\mathcal {T},\delta )$. So, consider the competency *A* and the bag $\mathcal {B}^A$ selected at iteration 0≤k<l. Then *A* distinguishes some pair $\{C, C'\} \in \mathcal {D}^-_k$ which is not distinguished by any competency in $\mathcal {T}_k$, meaning that competency *A* is necessary at iteration *k*. For the step from *k* to k+1 then define $\mathbb {B}_{k+1} = \{\mathcal {B}\setminus (\mathcal {B}^A\setminus \{A\}):\mathcal {B}\in \mathbb {M}_k\}$. This does not empty any bag in $\mathbb {M}_{k}$, and the only bag in $\mathbb {M}_{k+1}$ which can distinguish the pair of competence states $\{C, C'\}$ is the singleton bag $\{A\}$. Thus, $\{C, C'\} \in \mathcal {D}^+_{k+1}$, and $A \in \mathcal {T}_{k+1}$ remains necessary in all subsequent iterations, and cannot be deleted or replaced by any other competency. This completes the proof that $\mathcal {T}_l$ is a reduct.□

The following elaborates on the arguments in the proof and comments on their implications for the functioning of the CAP. Any competency included in a reduct is necessary to distinguish some pair $$\{C,C'\} \in \mathcal {D}$$ (equivalently, $$\{C,C'\} \in \mathcal {D}^\circ $$). Competency *A* selected from $$\mathcal {A}_k$$, which at *k* is not yet necessary to distinguish any pair, is made necessary by removing from each bag in $$\mathbb {M}_k$$ all competencies in the selected bag $$\mathcal {B}^A$$ except *A* (i.e., the elements of $$\mathcal {B}^A \setminus \{A\}$$). This operation ensures that *A* becomes necessary to distinguish some pair (specifically, any pair $$\{C,C'\} \in \mathcal {D}$$ with $$\delta (C,C') \cap \bigcup \mathbb {M}_k = \mathcal {B}^A$$), and that none of the competencies in $$\mathcal {B}^A \setminus \{A\}$$ remain available for selection in any later iteration. Consequently, *A* will be included in the resulting reduct, whereas none of the competencies in $$\mathcal {B}^A \setminus \{A\}$$ will be. We note that, once *A* is in the reduct, it can be used to distinguish every pair $$\{C,C'\} \in \mathcal {D}$$ whose bag $\delta (C,C^{\prime })$ contains *A*. Moreover, since no bag in $$\mathbb {M}_k$$ is a proper subset of any other, $$\mathcal {B}^A \setminus \{A\}$$ can be subtracted from each bag in $$\mathbb {M}_k$$ without emptying any of them (i.e., without losing the possibility of distinguishing some pair $$\{C,C'\} \in \mathcal {D}$$).

The collection $\mathcal {T}_k = \bigcup \{\mathcal {B}\in \mathbb {M}_k:|\mathcal {B}|=1\}$ contains all competencies that, at iteration *k*, are necessary to distinguish some pair $\{C,C'\}\in \mathcal {D}$. At k=0, $\mathcal {T}_0$ equals the core $\mathcal {T}^c$ of $(S,\mathcal {D},\mathcal {T},\delta )$ (5), that is, the set of competencies in $\mathcal {T}$ that are included in every reduct of $(S,\mathcal {D},\mathcal {T},\delta )$. The discernibility system $(S,\mathcal {D},\mathcal {T}_0,\delta _0)$ may already be a reduct; otherwise, it is a partial reduct. In the former case, the CAP stops at k=0. In the latter case, starting from $\mathcal {T}_0 = \mathcal {T}^c$, the competencies that become necessary are added to $\mathcal {T}_k$ at each iteration *k* until a reduct of $(S,\mathcal {D},\mathcal {T},\delta )$ is obtained. Specifically, these are the competency $A \in \mathcal {A}_k$ selected for addition and any other competency that occurs as the sole element of some bag after deleting $\mathcal {B}^A \setminus \{A\}$ (i.e., any competency $B \in \mathcal {A}_k$, B≠A, such that, for some $\mathcal {B}\in \mathbb {M}_k$, $\mathcal {B}\setminus (\mathcal {B}^A \setminus \{A\}) = \{B\}$).

If the CAP stops at k=l, $(S,\mathcal {D},\mathcal {T}^\diamond ,\delta ^\diamond )$ denotes the resulting reduct of $(S,\mathcal {D},\mathcal {T},\delta )$, where $\mathcal {T}^\diamond = \bigcup \mathbb {M}_{l}$. The following example shows a run of the CAP in which *A* and $\mathcal {B}^A$ are selected at random.

### Example 9

Consider again the discernibility system $(S,\mathcal {D},\mathcal {T},\delta )$ in Example 5, with bag set $\mathbb {B}$. Table 5 illustrates a possible run of the CAP. The minimal bag set $\mathbb {M}_k$ and the collection $\mathcal {A}_k$ of competencies that can be added are shown, whereas, for the sake of space, the bag set $\mathbb {B}_k$ is omitted. Suppose that, at iteration *k*, the bolded competency in $\mathcal {A}_k$ is randomly selected for addition. Suppose also that, among all bags including the selected competency (which are underlined), the bolded one is randomly selected. Then, the procedure stops at k=2 (where $\mathcal {A}_2 = \emptyset$) and leads to the reduct $(S,\mathcal {D},\mathcal {T}^\diamond ,\delta ^\diamond )$ of $(S,\mathcal {D},\mathcal {T},\delta )$, with competency set $\mathcal {T}^\diamond = \bigcup \mathbb {M}_2 = \{b,ab,ac,cd\}$. Among the four competencies in $\mathcal {T}^\diamond$, *b* is already necessary because it is a core competency, *cd* and *ab* become necessary when they are selected for addition at k=0 and 1, respectively, and *ac* becomes necessary at k=1 when $\{a,ab\}\setminus \{ab\} = \{a\}$ is deleted from $\{a,ac\}$, leaving $\{ac\}$ as a singleton bag. It is worth noting that, to construct the same reduct, the CDP satisfied the termination criterion at k=5 (Example 5).

The remainder of this section compares the CDP and the CAP. The CDP obtains a reduct of the discernibility system $(S,\mathcal {D},\mathcal {T},\delta )$ by iteratively removing competencies from the full competency set $\mathcal {T}$. Conversely, the CAP obtains a reduct by iteratively adding competencies to the core $\mathcal {T}^c$ of $(S,\mathcal {D},\mathcal {T},\delta )$. Thus, the CDP and the CAP seek to obtain a reduct of $(S,\mathcal {D},\mathcal {T},\delta )$ from simplifications and partial reducts of $(S,\mathcal {D},\mathcal {T},\delta )$, respectively. In both procedures, competencies are removed from $\mathcal {T}$ or added to $\mathcal {T}^c$ only if the current set does not already define a reduct of the discernibility system.

**Table 6:** Run of the competency addition procedure in Example 11, with selection of competencies for addition and bags based on the preference relation in Eq. 4

*k*	$\mathbb {M}_k$	$\mathcal {A}_k$
0	$\{\{b\},\{a,ab\},\{a,c,ac\}$,$\{c,ac,bc,abc\}$, $\{c,bc,bd,cd,bcd\}$, $\underline{\boldsymbol{\{ad,bd,cd,abd,acd,bcd,abcd\}}}\}$	$\{a,c,ab,ac,\underline{\boldsymbol{ad}},bc,bd,cd,\underline{abc},abd,acd,bcd,abcd\}$
1	$\{\{b\},\{a,ab\},\underline{\underline{\boldsymbol{\{a,c,ac\}}}},\underline{\{c,bc\}},\{ad\}\}$	$\{a,\underline{\boldsymbol{c}},ab,ac,\underline{bc}\}$
2	$\{\{b\},\{ab\},\{c\},\{ad\}\}$	∅

*Note.* Most preferred competencies are underlined, and selected competencies are in bold. Bags containing selected competencies are underlined, least preferred bags are underlined twice, and selected bags are in bold

To construct the reduct $(S,\mathcal {D},\mathcal {T}^\diamond ,\delta ^\diamond )$ of $(S,\mathcal {D},\mathcal {T},\delta )$, the CDP iteratively removes from $\mathcal {T}$ the competencies that are not necessary to distinguish any pair $\{C,C'\} \in \mathcal {D}$. At each iteration, the competency selected for deletion is removed, together with any other competency that occurs only in non-minimal bags. Hence, the procedure stops after at most $|\mathcal {T}|-|\mathcal {T}^\diamond |$ iterations (i.e., $l_\text {CDP} \le |\mathcal {T}|-|\mathcal {T}^\diamond |$). To construct the same reduct, the CAP iteratively makes non-core competencies necessary to distinguish some pair $\{C,C'\} \in \mathcal {D}$. At each iteration, the competency selected for addition is made necessary, together with any other competency that occurs as the sole element of some bag after removing the competencies in the chosen bag except the selected one. Hence, the procedure stops after at most $|\mathcal {T}^\diamond |-|\mathcal {T}^c|$ iterations (i.e., $l_\text {CAP} \le |\mathcal {T}^\diamond |-|\mathcal {T}^c|$). For each of the 34 reducts of the discernibility system in Example 5, whose core is $\mathcal {T}^c=\{b\}$, Table 3 reports the iteration at which each procedure stops. In this example, the CDP always requires more iterations than the CAP. In general, however, no direct relation between $l_\text {CAP}$ and $l_\text {CDP}$ follows from the bounds above.

## Incorporating preferences into the competency addition procedure

When alternative competencies and bags containing them are eligible for selection, the choice among them can be guided by test developer preferences. For example, if items requiring more skills are preferred to those requiring fewer skills, one may favor including competencies involving more skills in the reduct and excluding competencies involving fewer skills. Similarly, if estimates of the careless error and lucky guess probabilities of the items are available, one may favor including competencies associated with items with lower probabilities and excluding competencies associated with items with higher probabilities. This section describes how test developer preferences can be incorporated into the CAP.

The preferred between any two competency sets $\mathcal {T}_1$ and $\mathcal {T}_2$, which are subsets of $\mathcal {T}$, can be determined based on the preference relation ≿ on the competencies in $\mathcal {T}$. This is achieved as follows. First, the competencies in $\mathcal {T}_1$ and those in $\mathcal {T}_2$ are ordered linearly based on ≿. Then, $\mathcal {T}_1$ and $\mathcal {T}_2$ are ordered by sequentially comparing the competencies they contain, one by one. In the left-to-right lexical order (Yao et al., 2006), $\mathcal {T}_1$ and $\mathcal {T}_2$ are compared from left to right to determine which competency set is preferred.

### Definition 3

(Left-to-right lexical order). Consider a weak order ≿ on the competency set $\mathcal {T}$. Given two nonempty subsets $\mathcal {T}_1$ and $\mathcal {T}_2$ of $\mathcal {T}$ (with *m* and *n* elements), let $T_{1,1} \succsim ... \succsim T_{1,m}$ and $T_{2,1} \succsim ... \succsim T_{2,n}$ denote the weak orders induced by ≿ on $\mathcal {T}_1$ and $\mathcal {T}_2$, respectively. $\mathcal {T}_1$ is said to be *preferred to* $\mathcal {T}_2$ *under the left-to-right lexical order*, denoted as $\mathcal {T}_1 \gg _\text {L2R} \mathcal {T}_2$, if and only if there exists $1 \le i \le \min \{m,n\}$ such that $T_{1,j} \sim T_{2,j}$ for 1≤j<i and $T_{1,i} \succ T_{2,i}$.

### Example 10

Consider the competency set $\mathcal {T}$ in Example 5 and the preference relation ≿ on $\mathcal {T}$ given in Eq. 4. Consider also the two subsets $\mathcal {T}_1 = \{b,cd,abc,bcd\}$ and $\mathcal {T}_2=\{a,ad,bc,bd,acd\}$ of $\mathcal {T}$. The elements of $\mathcal {T}_1$ and $\mathcal {T}_2$ are ordered under ≿ as

$$ abc \succ cd \sim bcd \succ b $$

and

$$ ad \succ bc \succ a \sim acd \succ bd, $$

respectively. $\mathcal {T}_1$ is preferred to $\mathcal {T}_2$ under the left-to-right lexical order. Indeed, by Eq. 4, the first two positions satisfy abc∼ad, cd∼bc, whereas at the third position bcd≻a.

It is easy to verify that, with $\mathbb {T}\subseteq 2^{\mathcal {T}}$ being a collection of subsets of $\mathcal {T}$, $\gg _\text {L2R}$ establishes a strict weak order (negatively transitive and asymmetric relation) on $\mathbb {T}$. A competency set $\mathcal {T}_1 \in \mathbb {T}$ is said to be *most preferred* under the strict weak order $\gg _\text {L2R}$ if there is no $\mathcal {T}_2 \in \mathbb {T}$ such that $\mathcal {T}_2 \gg _\text {L2R} \mathcal {T}_1$. A competency set $\mathcal {T}_1 \in \mathbb {T}$ is said to be *least preferred* under $\gg _\text {L2R}$ if there is no $\mathcal {T}_2 \in \mathbb {T}$ such that $\mathcal {T}_1 \gg _\text {L2R} \mathcal {T}_2$. Stating that a competency set is most preferred under the left-to-right lexical order $\gg _\text {L2R}$ means that the set contains the largest number of competencies that are most preferred, second most preferred, and so on, according to the preference relation ≿. Stating that a competency set is least preferred under $\gg _\text {L2R}$ means that the set contains the smallest number of competencies that are most preferred, second most preferred, and so on, according to ≿.

An *add most preferred* (AMP) rule is defined that selects any competency in $\mathcal {A}_k$ that is most preferred (i.e., leftmost) based on ≿. If more than one competency in $\mathcal {A}_k$ satisfies this criterion, one of them is chosen at random.

Suppose *A* is the competency selected for addition at iteration *k*, and $\mathbb {M}^A_k = \{\mathcal {B}\in \mathbb {M}_k:A\in \mathcal {B}\}$ is the set of all minimal bags containing *A* at *k*. If $\mathbb {M}^A_k$ contains a single bag, then all competencies in that bag except *A* are removed from all bags in $\mathbb {M}_k$. If $\mathbb {M}^A_k$ contains more than one bag, then one of them is selected, and all competencies in it except *A* are removed from all bags in $\mathbb {M}_k$. Specifically, the bag $\mathcal {B}^A$ is selected for which the set $\mathcal {B}^A \setminus \{A\}$ is least preferred under $\gg _\text {L2R}$. If more than one bag in $\mathbb {M}^A_k$ satisfies this criterion, one of them is chosen at random. The reason for removing the competencies in the least-preferred set $\mathcal {B}^A \setminus \{A\}$ is to try to keep the most-preferred competencies available for potential inclusion in the reduct.

Given the discernibility system $(S,\mathcal {D},\mathcal {T},\delta )$, let $\mathbb {T}^\diamond$ denote the collection of all subsets $\mathcal {T}^\diamond$ of $\mathcal {T}$ such that $(S,\mathcal {D},\mathcal {T}^\diamond ,\delta ^\diamond )$ is a reduct of $(S,\mathcal {D},\mathcal {T},\delta )$. A reduct $(S,\mathcal {D},\mathcal {T}^\diamond ,\delta ^\diamond )$ is said to be most preferred under ≫ if its competency set $\mathcal {T}^\diamond \in \mathbb {T}^\diamond$ is most preferred under ≫. The CDP strives to exclude the least preferred competencies and the reducts produced by this procedure are most preferred under the right-to-left lexical order $\gg _\text {R2L}$ (Anselmi et al., 2024b). Even if the CAP strives to include the most preferred competencies, the reducts produced by this procedure need not be most preferred under the left-to-right lexical order $\gg _\text {L2R}$. Intuitively, the deletion of $\mathcal {B}^A \setminus \{A\}$ and the construction of $\mathbb {B}_{k+1}$ from $\mathbb {M}_k$ (Step 4 of the CAP) may exclude competencies belonging to the $\gg _\text {L2R}$-most-preferred reducts. If such competencies belong to $\bigcup (\mathbb {B}_k \setminus \mathbb {M}_k)$ at any iteration *k*, they become unavailable for selection in subsequent iterations and cannot be included in the reduct. Conversely, a less-preferred competency may be included in the reduct if it becomes necessary (i.e., it occurs as the sole element of some bag) after the deletion of $\mathcal {B}^A \setminus \{A\}$.

### Example 11

Consider the discernibility system $(S,\mathcal {D},\mathcal {T},\delta )$ in Example 5 and the preference relation ≿ on $\mathcal {T}$ in Eq. 4. Table 6 illustrates a possible run of the CAP based on this preference relation. Suppose that, at iteration *k*, the bolded competency in $\mathcal {A}_k$ is selected for addition among the most-preferred competencies (which are underlined). Suppose also that, among the least-preferred bags including the selected competency (which are underlined), the bolded bag in $\mathbb {M}_k$ is selected. Then, the procedure stops at k=2 (where $\mathcal {A}_2 = \emptyset$) and leads to the reduct $(S,\mathcal {D},\mathcal {T}^\diamond ,\delta ^\diamond )$ of $(S,\mathcal {D},\mathcal {T},\delta )$, with competency set $\mathcal {T}^\diamond = \bigcup \mathbb {M}_2 = \{b,ab,c,ad\}$. Four reducts of $(S,\mathcal {D},\mathcal {T},\delta )$ are $\gg _\text {L2R}$-most preferred, namely those with competency sets $\mathcal {T}^\diamond _6$, $\mathcal {T}^\diamond _9$, $\mathcal {T}^\diamond _{17}$, and $\mathcal {T}^\diamond _{20}$ (see Table 3). Each includes one most-preferred competency, one second-most-preferred competency, and two third-most-preferred competencies, whereas the remaining 30 reducts include fewer such high-ranked competencies. Running the CAP under Eq. 4 yields the four reducts with competency sets $\mathcal {T}^\diamond _3$, $\mathcal {T}^\diamond _6$, $\mathcal {T}^\diamond _9$, and $\mathcal {T}^\diamond _{20}$. This example shows that the CAP-generated reducts are not always $\gg _\text {L2R}$-most preferred. In constructing the reduct with competency set $\mathcal {T}^\diamond _3$, which is exactly the one obtained above, deleting $\{a,c,ac\}\setminus \{c\}=\{a,ac\}$ from all bags in $\mathbb {M}_1$ removes *a* – one of the third-most-preferred competencies – and makes *ab* – one of the fourth-most-preferred competencies – necessary (see Table 6). Consequently, $\mathcal {T}^\diamond _3$ contains one third-most-preferred competency and one fourth-most-preferred competency, rather than two third-most-preferred competencies. The example also shows that the CAP does not necessarily produce all $\gg _\text {L2R}$-most-preferred reducts. For example, the reduct with competency set $\mathcal {T}^\diamond _{17}$ is not obtained by running the CAP based on Eq. 4.

## Real-life applications

The applications presented in this section pertain to the assessment of arithmetic skills, which are fundamental mathematical abilities necessary for performing basic operations with numbers and essential for everyday problem solving and decision-making. Anselmi et al. (2017) considered six skills involved in solving arithmetic items in primary school, namely: (*a*) Addition without carrying, (*b*) Subtraction without column borrowing, (*c*) Addition with carrying, (*d*) Subtraction with column borrowing, (*e*) Multiplication, and (*f*) Division. They also assumed that skill *a* is a prerequisite of skill *c*, and that skill *b* is a prerequisite of skill *d*. This means that *c* cannot be mastered without mastering *a*, and *d* cannot be mastered without mastering *b*. Applying the considered prerequisite relations to the collection $S = \{a,b,...,f\}$ of the six skills results in the competence structure

6 $$\begin{aligned} \mathcal {C}=&\{\emptyset ,a,b,e,f,ab,ac,ae,af,bd,be,bf,ef, abc,abd, \nonumber \\&abe,abf,ace,acf,aef,bde,bdf,bef,abcd,abce, \nonumber \\&abcf,abde,abdf,abef,acef,bdef,abcde,abcdf,\nonumber \\&abcef,abdef,abcdef\}, \end{aligned}$$

which is used throughout this section. It should be noted that, consistent with the defined prerequisite relations, any competence state that includes *c* also includes *a*, and any competence state that includes *d* also includes *b*. $$\mathcal {C}$$ consists of 36 competence states and is a *well-graded competence space*, that is, a competence structure closed under union (for any two competence states *C* and $C^{\prime }$ in $$\mathcal {C}$$, their union $C\cup C^{\prime }$ is also a competence state in $$\mathcal {C}$$) and downgradable (for every nonempty competence state *C* in $$\mathcal {C}$$, there exists a skill *s* in *C* such that removing *s* from *C* yields another competence state in $$\mathcal {C}$$). Well-graded competence spaces are particularly interesting from a pedagogical point of view as they allow smooth learning: whatever the competence state of the individual, they can learn one new skill at a time (Cosyn & Uzun, 2009).

In this section, the CAP is used to construct from scratch a test that assesses the six skills in *S* and to improve and shorten an existing test that measures these skills. It is assumed that items can be constructed for any nonempty subset of *S* and that items requiring more skills to be solved are preferred to items requiring fewer. These assumptions are made for illustrative purposes only and are in no way necessary.

### Constructing a test from scratch

We want to construct a fully informative test, that is, a test that allows for uniquely uncovering the competence state of any student based on their knowledge state. Since the test should be capable of distinguishing any competence state in $$\mathcal {C}$$ from the others, $$\mathcal {D}$$ is set to be equal to the collection  of all 630 unordered pairs of the 36 competence states in $$\mathcal {C}$$. Assuming that items can be constructed for any nonempty subset of *S*, $$\mathcal {T}$$ is set to be equal to the collection $$2^S \setminus \{\emptyset \}$$ of all 63 nonempty subsets of *S*. The discernibility system $$(S, \mathcal {D}, \mathcal {T}, \delta )$$ is considered in the construction of the test. Assuming that items requiring more skills are preferred to those requiring fewer, the preference relation ≿ on $$\mathcal {T}$$ is defined in terms of the number of skills in each competency as follows: for all $$T, T' \in \mathcal {T}$$, $T≿T^{\prime }$ if and only if $\left|T|\geq \right|T^{\prime }|$.

With $\mathbb {B}$ being the bag set of $(S,\mathcal {D},\mathcal {T},\delta )$, the initial bag set is set to be $\mathbb {B}_0 = \mathbb {B}$. The CAP based on ≿ produces the reduct $(S, \mathcal {D}, \mathcal {T}^\diamond , \delta ^\diamond )$ of $(S, \mathcal {D}, \mathcal {T}, \delta )$, with competency set $\mathcal {T}^\diamond =\{a,b,e,f,ac,bd\}$. There are four reducts of $(S,\mathcal {D}, \mathcal {T}, \delta )$, with the other three having competency sets $\mathcal {T}^\diamond _1=\{a,b,c,d,e,f\}$, $\mathcal {T}^\diamond _2=\{a,b,c,e,f,bd\}$, and $\mathcal {T}^\diamond _3=\{a,b,d,e,f,ac\}$. Among these, the reduct produced by the CAP is the most preferred under the left-to-right lexical order $\gg _\text {L2R}$ based on ≿.

The competency set $\mathcal {T}^\diamond = \{a,b,e,f,ac,bd\}$ specifies the exact competencies that the test items must cover. By constructing a test *Q* such that for each of the six competencies $T \in \mathcal {T}^\diamond$ there is an item q∈Q with $\mu (q) = \{T\}$, a fully informative conjunctive competence model $(Q,S,\mathcal {C},\mu )$ is obtained. Furthermore, if *Q* contains exactly one item for each competency, $(Q,S,\mathcal {C},\mu )$ is also minimal. Table 7 presents an example of a six-item arithmetic test that can be validated using the methods outlined in Section “Competence models”. The six-item test containing one item for each of the six competencies in $\mathcal {T}^\diamond$ is as informative for identifying competence states from knowledge states as a hypothetical 63-item test containing one item for each of the 63 competencies in $\mathcal {T}$.

This example shows that, when constructing a test from scratch, the proposed method identifies the exact competencies that the items must cover for the test to be as informative as possible. Because it identifies a minimal set of competencies, test length is kept minimal.

### Improving an existing test

Anselmi et al. (2017) considered a test consisting of 12 open-response arithmetic items, which were assumed to require the six skills in $S = \{a,b,...,f\}$ to be solved. The conjunctive competence model $(Q,S,\mathcal {C},\mu )$ is considered, where $\mathcal {C}$ is the competence structure in Eq. 6 and $Q = \{1,2,...,12\}$ is the test consisting of 12 items that are associated with the six skills in *S* as follows:

7 $$\begin{aligned} \begin{array}{llll} \mu (1)=\{abcd\}, & \mu (2)=\{aef\}, & \mu (3) = \{aef\}, & \mu (4)=\{abcde\},\\ \mu (5)=\{abcd\}, & \mu (6)=\{bde\}, & \mu (7) = \{abce\}, & \mu (8)=\{f\},\\ \mu (9)=\{e\}, & \mu (10)=\{acf\}, & \mu (11) = \{af\}, & \mu (12)=\{acf\}. \end{array} \end{aligned}$$

Item 3 is given as an example: “Andrew writes 12 pages an hour and goes on writing for 6 hours. He then decides to arrange them on 8 shelves. How many pages are on each shelf?” The assumption was made that the item requires skills *a* (“Addition without carrying”), *e* (“Multiplication”), and *f* (“Division”). The competence model $(Q,S,\mathcal {C},\mu )$ has been validated on empirical data from 129 primary school children with satisfactory results (Anselmi et al., 2025a). However, the 36 competence states in $\mathcal {C}$ delineate only 17 distinct knowledge states, meaning that $(Q,S,\mathcal {C},\mu )$ is not fully informative.

We want to add a minimal set of items to test *Q* so that the resulting test is fully informative about the underlying competence states. This entails seeking a domain extension $$(Q^+,S,\mathcal {C},\mu ^+)$$ of $$(Q,S,\mathcal {C},\mu )$$ that is fully informative, with each added item contributing to its full informativeness. To achieve this, the discernibility system $$(S,\mathcal {D},\mathcal {T},\delta )$$ is considered, where $$S = \{a,b,...,f\}$$, $$\mathcal {D}$$ is set to be equal to the collection  of all 48 unordered pairs of competence states in $$\mathcal {C}$$ that, via competence model $$(Q,S,\mathcal {C},\mu )$$, delineate the same knowledge state (i.e., all pairs of competence states that cannot be distinguished by the competencies associated with the items in *Q*), and $$\mathcal {T}$$ is set to be equal to the collection $$2^S\setminus (\{\emptyset \} \cup \bigcup _{q \in Q}\mu (q))$$ of all 54 nonempty competencies in $$2^S$$ that are not associated with any item in *Q*. The definition of $$\mathcal {T}$$ becomes clear when considering that competencies not already associated with the items in *Q* must be evaluated to distinguish pairs of competence states that items in *Q* cannot distinguish, and that items are assumed to be constructible for any nonempty subset of *S*. The preference relation ≿ on $$\mathcal {T}$$ assumes that items requiring more skills are preferred over those requiring fewer skills: for all $$T, T' \in \mathcal {T}$$, $T≿T^{\prime }$ if and only if $\left|T|\geq \right|T^{\prime }|$.

**Table 7:** Example of an arithmetic test developed from scratch

q∈Q		μ(q)
1	Mary has 2 colored pencils in one pencil case and 5 colored pencils in another pencil case. How many colored pencils does she have in total?	*a*
2	A painter has 24 cans of paint. He used three of them to paint a fence. How many cans does he have left?	*b*
3	Julia eats three apples every day. Therefore, how many apples does she eat in 1 week?	*e*
4	John has 10 marbles and wants to distribute them equally to his two best friends. How many marbles will he give to each of them?	*f*
5	Mary has 23 euros in her piggy bank. In the morning, her mother puts 15 euros in the piggy bank. In the evening, her father puts 19 euros in the piggy bank. How many euros does Mary have in her piggy bank at the end of the day?	*ac*
6	Scarlett has 326 stamps and decides to give 15 of them to her little sister Francis. She later decides to give 15 to her little brother Anthony as well. How many stamps does she have left?	*bd*

*Note.* *a* = addition without carrying; *b* = subtraction without column borrowing; *c* = addition with carrying; *d* = subtraction with column borrowing; *e* = multiplication; *f* = division

With $\mathbb {B}$ being the bag set of $(S,\mathcal {D},\mathcal {T},\delta )$, the initial bag set is set to be $\mathbb {B}_0 = \mathbb {B}$. The CAP based on ≿ yields $(S, \mathcal {D}, \mathcal {T}^\diamond , \delta ^\diamond )$, with competency set $\mathcal {T}^\diamond =\{a,b,ac,bd\}$. This is one of the four reducts of $(S,\mathcal {D}, \mathcal {T}, \delta )$, the other three having competency sets $\mathcal {T}^\diamond _1=\{a,b,c,d\}$, $\mathcal {T}^\diamond _2=\{a,b,c,bd\}$, and $\mathcal {T}^\diamond _3=\{a,b,d,ac\}$. The reduct produced by CAP is the most preferred under the left-to-right lexical order $\gg _\text {L2R}$ based on ≿.

The collection $\mathcal {T}^\diamond = \{a,b,ac,bd\}$ specifies the exact competencies that the items added to the test must cover. Extending $Q = \{1,2,...,12\}$ to $Q^+=\{1,2,...,16\}$ with $\mu ^+(13)=\{a\}$, $\mu ^+(14)=\{b\}$, $\mu ^+(15)=\{ac\}$, and $\mu ^+(16)=\{bd\}$ results in the fully informative competence model $(Q^+,S,\mathcal {C},\mu ^+)$. Each of the four added items contributes to making the test fully informative. Table 7 provides an example of four arithmetic items assumed to require the competencies in $\mathcal {T}^\diamond$ (items 1, 2, 5, and 6 in the table). These items can be validated using the methods outlined in Section “Competence models”. Adding these four items suffices to obtain an improved test that is as informative as a hypothetical test obtained by adding 54 items, one for each of the 54 competencies in $\mathcal {T}$.

This example shows that, when improving an existing test, the proposed method identifies the exact competencies that the added items must cover to maximize the gain in test informativeness. Because it identifies a minimal set of competencies, the increase in test length is also kept minimal.

### Shortening an existing test

We want to develop a minimal shortened form of the 12-item test *Q* in Section “Improving an existing test” that is as informative about individuals’ competence states as *Q*. Thus, if $(Q,S,\mathcal {C},\mu )$ is the conjunctive competence model including test *Q*, we seek a domain restriction $(Q^-,S,\mathcal {C},\mu ^-)$ of $(Q,S,\mathcal {C},\mu )$ that is as informative as $(Q,S,\mathcal {C},\mu )$ and minimal. To achieve this, the discernibility system $(S,\mathcal {D},\mathcal {T},\delta )$ is considered, where $S = \{a,b,...,f\}$, $\mathcal {D}$ is set to be equal to the collection  of all 630 unordered pairs of the competence states in $\mathcal {C}$,^2^ and $\mathcal {T}$ is set to be equal to the collection $\bigcup _{q \in Q} \mu (q)$ of all nine competencies associated with the 12 items in *Q* Eq. 7, that is

$$ \mathcal {T}= \{e,f,af,acf,aef,bde,abcd,abce,abcde\}. $$

The bag set $\mathbb {B}$ of $(S,\mathcal {D},\mathcal {T},\delta )$ is used as initial bag set $\mathbb {B}_0$. The CAP based on ≿ yields the reduct $(S, \mathcal {D}, \mathcal {T}^\diamond , \delta ^\diamond )$ of $(S,\mathcal {D}, \mathcal {T}, \delta )$, with $\mathcal {T}^\diamond =\{e,f,af,acf,bde,abcd,abce\}$. Since $\mathcal {T}^\diamond$ equals the core $\mathcal {T}^c$ of $(S,\mathcal {D}, \mathcal {T}, \delta )$, it is the unique reduct of $(S,\mathcal {D}, \mathcal {T}, \delta )$ (Section “Partial reduct and core”) and is obtained by the procedure at iteration k=0.

The competency set $\mathcal {T}^\diamond = \{e,f,af,acf,bde,abcd,abce\}$ is useful for selecting the items that the shortened test form must include. This implies that seven items are sufficient to assess the underlying competence states as the original 12-item test. A domain restriction $(Q^-,S,\mathcal {C},\mu ^-)$ of $(Q,S,\mathcal {C},\mu )$ that is as informative as $(Q,S,\mathcal {C},\mu )$ and minimal is obtained by selecting seven items from the 12 in *Q*, one for each competency in $\mathcal {T}^\diamond$. The test *Q* contains two items for each of the competencies *acf* (items 10 and 12) and *abcd* (items 1 and 5) and one item for each of the remaining five competencies in $\mathcal {T}^\diamond$ (items 6, 7, 8, 9, and 11; Eq. 7). Therefore, the shortened test form must include items 6, 7, 8, 9, and 11, plus one item chosen from items 10 and 12 and another item chosen from items 1 and 5. The selection could be based on the careless error and lucky guess probabilities of the items. Specifically, to increase the chances of correctly uncovering students’ competence states from their item responses, the item in each pair with the lower sum of the careless error and lucky guess probabilities should be chosen (Heller & Repitsch, 2012) . These sums are .337, .208, .500, and .068 for items 1, 5, 10, and 12, respectively (Table 3 in Anselmi et al., 2025a). Thus, items 12 and 5 are selected from the respective pairs. The resulting short test is $Q^-=\{5,6,7,8,9,11,12\}$. Any alternative shortened test form containing more than seven items but lacking at least one of the seven competencies in $\mathcal {T}^\diamond$ would be less informative than the constructed shortened test form.

This example shows that, when shortening an existing test, the proposed method identifies the exact competencies that the retained items must cover so that the shortened test form is as informative as the original test. Because it identifies a minimal set of competencies, the length of the shortened test form is kept minimal.

## Final remarks

This work introduces a competency addition procedure (CAP) for constructing reducts of a discernibility system and demonstrates its real-world use in building a test from scratch and in improving and shortening an existing test. This section elaborates on extensions of the presented approach and outlines directions for future research.

The duality between conjunctive and disjunctive models allows the methodology developed here for conjunctive tests to be applied to the construction, improvement, and shortening of disjunctive tests (Anselmi et al., 2022; Anselmi et al., 2024b). When applying the CAP, we consider a competence structure $\mathcal {C}$ on *S* and the collection  of all pairs of competence states $C,C'\in \mathcal {C}$ that must be distinguished. To construct, improve, or shorten a disjunctive test, the CAP is applied to the discernibility system $(S,\mathcal {D}^\partial ,\mathcal {T},\delta )$, where $\mathcal {D}^\partial =\{\{S\setminus C,S\setminus C'\}:\{C,C'\}\in \mathcal {D}\}$ is obtained by replacing each pair in $\mathcal {D}$ with the corresponding pair of complementary competence states with respect to *S*, and $\mathcal {T}$ is the collection of all nonempty subsets T⊆S such that an item that can be solved using any of the skills in *T* is available or can be constructed. Thus, the CAP itself remains unchanged, while the discernibility system is replaced by its dual counterpart. A preference relation ≿ on the competencies in $\mathcal {T}$ can be defined as in the present work. In the disjunctive model, possessing any of the skills associated with an item is sufficient for mastering it. Thus, for any two competencies $T_1,T_2 \in \mathcal {T}$, $T_1 \succsim T_2$ means that an item that can be mastered using any of the skills in $T_1$ is preferred to an item that can be mastered using any of the skills in $T_2$.

By defining a preference relation ≿ on $\mathcal {T}$, it is possible to develop tests with specific characteristics. Anselmi et al. (2024b) outlined some viable options for defining ≿, which include preferring competencies with many or few skills, those included in many bags, or those associated with items that have low careless error and lucky guess probabilities. The first option has been used in the applications of the CAP illustrated in Section “Real-life applications”. Additionally, the authors showed how these and other preference relations can be combined to form new preference relations. Two aspects of the CAP based on ≿ are discussed. First, the CAP does not always yield the most preferred reducts under the left-to-right lexical order, though it may do so under certain conditions. For instance, it generated reducts that were not the most preferred under the left-to-right lexical order based on ≿ in Example 11, whereas it produced only the most preferred reducts in the real-life applications. Future work could explore the conditions under which the CAP generates reducts most preferred under the left-to-right lexical order. Second, in this work, the left-to-right lexical order has been used to select the least-preferred competencies for exclusion from the reduct under construction. This choice is reasonable because the same order has been used to determine the preferred reducts. Alternatively, the right-to-left lexical order could be used to select the least-preferred competencies for exclusion. Future work could investigate how these two orders affect reduct generation.

The CAP presented in this work, together with the CDPs described in Anselmi et al. (2022) and Anselmi et al. (2024b), assumes that the competencies for which items are available or can be developed are known in advance. This assumption is easily satisfied when shortening an existing test because the relevant competencies are those already associated with the test items. By contrast, it may be unrealistic when constructing a test from scratch or improving an existing test. In such cases, items requiring specific skills need to be developed, and some may be difficult or even impossible to create. Future work could focus on integrating the CAP with a query procedure that iteratively asks experts whether they can provide items requiring specific skills and terminates once further queries would no longer lead to a more informative test. The integrated procedure would then be applied to the construction and improvement of tests.

## Data Availability

Not applicable.

## Appendix

**Table:**

Concept	Description
Discernibility system$(S,\mathcal {D},\mathcal {T},\delta )$	System that, for each pair of competence states, specifies which competencies distinguish that pair.
Bag $\delta (C,C^{\prime })$	Collection of competencies, each of which is sufficient to distinguish some pair of competence states.
Simplification $(S,\mathcal {D},\mathcal {T}^\diamond ,\delta ^\diamond )$	System whose competency set is a subset of the original competency set and distinguishes exactly the same pairs of competence states as the original competency set.
Reduct $(S,\mathcal {D},\mathcal {T}^\diamond ,\delta ^\diamond )$	System whose competency set is a minimal subset, with respect to set inclusion, of the original competency set and distinguishes exactly the same pairs of competence states as the original competency set.
Partial reduct $(S,\mathcal {D},\mathcal {T}^\bullet ,\delta ^\bullet )$	System whose competency set is a strict subset of the competency set of some reduct of a discernibility system; it does not distinguish all pairs of competence states distinguished by the original competency set.
Core $\mathcal {T}^c$	Set of all competencies that occur as the sole element of some bag. It equals the intersection of the competency sets of all reducts.
Competency deletion procedure	Iterative method that successively deletes competencies from the full competency set until the remaining set forms a reduct.
Competency addition procedure	Iterative method that successively adds competencies to the core until the resulting set forms a reduct.

## Footnotes

1 In previous work (Anselmi et al., 2022; 2024b), a reduct was equivalently defined as a simplification $(S,\mathcal {D},\mathcal {T}^\diamond ,\delta ^\diamond )$ of $(S,\mathcal {D},\mathcal {T},\delta )$ such that, for each $T \in \mathcal {T}^\diamond$, there is some pair $\{C,C'\} \in \mathcal {D}$ satisfying $\delta (C,C') \ne \emptyset$ and $((\mathcal {T}^\diamond \setminus \{T\}) \cap \delta (C,C') = \emptyset )$. The equivalence between the two definitions follows from the fact that, for any simplification, $\delta ^\diamond (C,C') = \delta (C,C') \cap \mathcal {T}^\diamond$, with $\delta ^\diamond (C,C') \ne \emptyset$ whenever $\delta (C,C') \ne \emptyset$. Thus, since $T \in \mathcal {T}^\diamond$, if $\delta (C,C') \ne \emptyset$ and $((\mathcal {T}^\diamond \setminus \{T\}) \cap \delta (C,C') = \emptyset )$, then $\delta ^\diamond (C,C') = \{T\}$. Conversely, if $\delta ^\diamond (C,C') = \{T\}$, then $\delta (C,C') \ne \emptyset$ and $((\mathcal {T}^\diamond \setminus \{T\}) \cap \delta (C,C') = \emptyset )$.

2 The bags of the 48 pairs of competence states in $\mathcal {C}$ that, via competence model $(Q,S,\mathcal {C},\mu )$, delineate the same knowledge state are empty. These 48 pairs can be excluded from $\mathcal {D}$ without affecting the results.

## Code Availability

A MATLAB function for constructing a reduct via the CDP in Section “Competency deletion procedure” and a script for running some examples are available at https://osf.io/p935x/?view_only=8661a330555f4e0da9adca8b4554371b (see Anselmi et al., 2024b, for details). A MATLAB function for constructing a reduct via the CAP in Section “Competency addition procedure”, as well as scripts for running some examples, constructing the test from scratch, and improving and shortening the existing test, are available at https://osf.io/85r4b/overview?view_only=01afc8d36d484b2aa31715d245903781.
